# Mechanism investigation and experiment validation of capsaicin on uterine corpus endometrial carcinoma

**DOI:** 10.3389/fphar.2022.953874

**Published:** 2022-09-21

**Authors:** Zhiheng Lin, Xiaohui Sui, Wenjian Jiao, Chong Chen, Xiaodan Zhang, Junde Zhao

**Affiliations:** ^1^ Shandong University of Traditional Chinese Medicine, Jinan, China; ^2^ Obstetrics Department of Affiliated Hospital of Weifang Medical College, Weifang, China; ^3^ Department of Traditional Chinese Medicine, Qilu Hospital of Shandong University, Jinan, China; ^4^ Shandong University Cheeloo College of Medicine Laboratory of Basic Medical Sciences, Jinan, China

**Keywords:** capsaicin, UCEC, mechanism investigation, experiment validation, bioinformatics

## Abstract

**Background:** Using bioinformatics analysis and experimental operations, we intend to analyze the potential mechanism of action of capsaicin target gene GATA1 in the treatment of uterine corpus endometrial carcinoma (UCEC) and develop a prognostic model for the disease to validate this model.

**Methods:** By obtaining capsaicin and UCEC-related DR-DEGs, the prognosis-related gene GATA1 was screened. The survival analysis was conducted via establishing high and low expression groups of GATA1. Whether the GATA1 could be an independent prognostic factor for UCEC, it was also validated. The therapeutic mechanism of capsaicin-related genes in UCEC was further investigated using enrichment analysis and immune methods as well as in combination with single-cell sequencing data. Finally, it was validated by cell experiments.

**Results:** GATA1, a high-risk gene associated with prognosis, was obtained by screening. Kaplan-Meier analysis showed that the survival of the high expression group was lower than that of low expression group. ROC curves showed that the prediction effect of the model was good and stable (1-year area under curve (AUC): 0.601; 2-years AUC: 0.575; 3-years AUC: 0.610). Independent prognosis analysis showed that the GATA1 can serve as an independent prognostic factor for UCEC. Enrichment analysis showed that “neuroactive Ligand - receptor interaction and TYPE I DIABETES MELLITUS” had a significant enrichment effect. Single-cell sequencing showed that the GATA1 was significantly expressed in mast cells. Cell experiments showed that the capsaicin significantly reduced the UCEC cell activity and migration ability, as well as inhibited the expression of GATA1.

**Conclusion:** This study suggests that the capsaicin has potential value and application prospect in the treatment of UCEC. It provides new genetic markers for the prognosis of UCEC patients.

## 1 Introduction

UCEC is a common cancer occurred in the female reproductive system, with a higher incidence in the group of menopausal women with a mean age of around 60 years (Paleari et al., 2021). According to the data statistics of American *Cancer* Association, about 65,950 UCEC patients will be newly diagnosed in the United States in 2022 (American Cancer Society, 2021). In recent years, due to the prevalence of aging and obesity, the incidence and mortality of UCEC are continuously increasing worldwide. The median survival of patients with recurrence and metastasis is also relatively low (McAlpine et al., 2016; Onstad et al., 2016). It not only seriously threatens the quality of life, health and survival of patients, but also increases the occupation of social medical resources. At present, the UCEC is mainly managed by surgical treatment, including total hysterectomy and bilateral salpingo-oophorectomy. Low-risk or medium-risk UCEC can also be managed by non-surgical treatment and adjuvant therapies such as radiotherapy and chemotherapy (Braun et al., 2016). However, the treatment method is still controversial for UCEC, including the use and evaluation of lymph node dissection and the options of adjuvant treatment. Besides, the choice of treatment directions is also relatively limited in advanced and metastatic tumors (van den Heerik et al., 2021; Brooks et al., 2019). In view of the increasing incidence and mortality of UCEC in recent years and the limited and controversial treatment options, it is very important to find a new treatment approach for UCEC and build a new prognostic model to accurately and conveniently predict the patient survival cycle.

Existing studies have found that the overall macro-regulatory effect of traditional Chinese medicine (TCM) has a significant effect on the control and prevention of diseases (Tang et al., 2008). Natural small molecule compounds in TCM have potential but not widely developed therapeutic effects in a variety of diseases, which not only play a role in primary health field in China currently, but also are widely used in the research on new cancer drugs (Zhang et al., 2012). Compared with traditional anticancer drugs with significant adverse effects, natural small molecule compounds of many traditional Chinese medicines are gradually attracting more and more attention due to their potential tumor selectivity and low cytotoxicity (Eritja et al., 2017). Capsaicin (trans-8-methyl-N-vanillyl-6-nonenamide) is a high-tech oxalic acid derivative. It is the main active compound of chili and participates in the formation of spicy taste (European Commission Health and Consumer Protection Directorate-General, 2002). It plays an important pharmacological role in anti-inflammation and analgesia, cardiovascular function protection, circadian regulation and anti-calculus, etc (Srinivasan, 2016; Lu et al., 2020). Recent studies have demonstrated that the capsaicin may be involved in regulating genes related to cancer cell growth and reproduction, survival, angiogenesis and metastasis, thus inhibiting tumor growth, as well as participate in the induction of apoptosis in various cancer cells including intestinal adenocarcinoma, pancreatic cancer and prostate cancer (Final report, 2007; Hanahan and Weinberg, 2011; Bley et al., 2012; Clark and Lee, 2016). Moreover, by searching literature, we have found that the capsaicin may also increase energy consumption, enhance lipid oxidation and reduce peripheral blood triglyceride levels in mice after intake (Josse et al., 2010; Lee et al., 2010; Kang et al., 2011). It is of great significance for controlling body weight and reducing the risk factors for the occurrence of UCEC. Moreover, there is a potential connection to the treatment of UCEC. The GATA1 is associated with erythropoiesis. It is a transcription factor that regulates cell cycle processes and may be involved in cell growth, differentiation and apoptosis (Kerenyi and Orkin, 2010; Crispino and Horwitz, 2017). Some studies have shown that the GATA1 is correlated with a poor prognosis of various female tumors (Boidot et al., 2010; Yang et al., 2021). Besides, it may promote the apoptosis of mast cells by knocking out GATA1, thereby controlling tumor progression to some extent (Blair et al., 1997; Migliaccio et al., 2003; Ribatti et al., 2005; Kitamura et al., 2006; Huang et al., 2008; Komi and Redegeld, 2020).

However, the role of capsaicin and its target gene GATA1 in UECC has not been further studied. In this context, we have used TCGA database transcriptome sequencing data analysis and single cell sequencing data analysis of GEO database to explore the potential mechanism and therapeutic target of capsaicin in the treatment of UCEC and build a new prognostic model. Finally, it is validated via cell experiments.

## 2 Methods

### 2.1 Acquisition of drug target gene datasets and differentially expressed genes of the disease

We searched the small molecule drug capsaicin through the Traditional Chinese Medicine Systems Pharmacology (TCMSP) database and obtained a total of 36 drug target proteins. The uniport (https://www.uniprot.org/) database was also used for gene conversion and standardization of target proteins to construct drug target gene datasets. We also searched The *Cancer* Genome Atlas (TCGA) website (https://portal.gdc.cancer.gov) and downloaded the gene expression quantification RNA-Seq (HTSeqFPKM) and transcription information of UCEC, as well as its clinical data. On the TCGA website, “corpus uteri” is listed as the primary site, and “TCGA” is listed as the program, and the options for “Disease Type” include “adenomas and adenocarcinomas” and “cystic, mucinous and serous neoplasms”. The other options are default. A total of 23 samples from the normal group and 552 samples from the tumor group samples were yielded. Data were extracted and standardized using R software (R 4.1.1). The Differentially expressed genes (DEGs) of treated samples were screened by the “limma” R package in the two groups. Screening parameter settings: *p* < 0.05, false discovery rate (FDR) < 0.05.

### 2.2 Acquisition and analysis of DR-differentially expressed genes

A total of 19 drug related DGEs (DR-DEGs) were obtained through taking the intersection of DEGs and drug target genes via Venny2.1.0 website (https://bioinfogp.cnb.csic.es/tools/venny/). We used the String (https://string-db.org/) website to build a protein interaction network of 19 DR-DEGs, and then imported the data into Cytoscape (3.9.0) for further analysis and visualization. The functional analysis was performed through its built-in “ClueGO” plugin.

### 2.3 Acquisition of prognosis-related DR-differentially expressed genes and validation

DRG-DEGs expression was combined with clinical data using the perl language (perl 5.30.0) and we removed samples with incomplete clinical data and a survival time of 0 or negative. We performed the univariate Cox regression analysis for the relationship between 19 DR-DEGs and survival through the coxph function of the “survival” R package. According to the analysis, we speculate that the GATA1 is more closely correlated with the prognosis of UCEC. In order to explore its relationship with GATA1 and other genes, we tested the correlation by pearsen and analyzed its size. According to the median value of GATA1 gene expression, the patients in the tumor group were divided into high and low expression groups. The overall survival (OS) was analyzed between the high and low expression groups, respectively. The results were presented by Kaplan-Meier curve. A time-dependent receiver operating characteristic (ROC) curve analysis was also performed using the “survivalROC” R package to test the specificity and sensitivity of the survival analysis. A new supplementary validation group has also been established. For the new supplementary validation group, we have selected 20% of the patients with the highest expression of GATA1 as the high GATA1 expression group (*n* = 107) and the top 20% of the patients with the lowest expression of GATA1 as the low GATA1 expression group (*n* = 107).

### 2.4 Clinical correlation and independent prognosis analysis

Paired tests were carried out for the normal and tumor tissues of the patients’ endometrium using the “limma” R package so as to explore GATA1’s difference in expression between the normal group and tumor group. To study the expression of GATA1 in different clinical ages and grade, as well as to assess whether the GATA1 expression, age and tumor grade could be used as independent prognostic indicators by both univariate and multivariate Cox regression analyses. In addition, we plotted the nomogram using “rms” R package to predict the outcome of each patient with UCEC and drew the calibration curves to compare and validate the accuracy of the nomograms for the 1,3 and 5-years survival prediction in UCEC patients.

### 2.5 Gene enrichment analysis

We used the “limma” R package to screen the DEGs from the high and low expression groups. Screening parameter settings: | logFCfilter |>1, false discovery rate (FDR) < 0.05. Kyoto Encyclopedia of Genes and Genomes (KEGG) enrichment analyses and Gene Ontology (GO) enrichment analyses were carried out for DEGs via the “clusterProfiler” R package. Expressed gene sets in the population of the high and low expression groups and tagged gene sets collected in Kyoto Encyclopedia of Genes and Genomes (KEGG) database V 7.5.1 were further analyzed using the “GSEA” R package. The statistical significance was defined as FDR<0.05.

### 2.6 Tumor microenvironment and immune correlation analysis

We calculated the immune scores, matrix scores and the total scores of the tumor microenvironment in the high and low GATA1 expression groups using the “estimate” R package. The scores of the immune cell infiltration in the high and low GATA1 expression groups were calculated by the CIBERSORT deconvolution algorithm. The correlation analysis was performed to screen the immune cells associated with GATA1 gene expression. Differences in expression of immune checkpoint-related genes were estimated by Wilcoxon test between the high and low GATA1 expression groups.

### 2.7 Using single-cell sequencing analysis to further validate the role of capsaicin target genes in uterine corpus endometrial carcinoma

We downloaded the single cell samples of 5 UCEC patients in the GSE173682 dataset from NCBI Gene Expression Omnibus (GEO) (https://www.ncbi.nlm.nih.gov/geo/), including GSM5276933, GSM5276934, GSM5276935, GSM5276936 and GSM5276937. They were also filtered using the R software (filtering condition settings: The number of transcript genes in a single cell is more than 200 and less than 7,500. The mitochondrial gene transcription volume is less than 25% and the total gene transcription volume is less than 100,000). These 34,210 cells filtered were given normalization using the NormalizeData function of the “Seurat” R package. The FindVariableFeatures function was used to further look for 3,000 hypervariable genes. Standardized scaling processing was carried out via the scaleData function. Cell stages were tested using the CellCycleScoring function. The dimensionality reduction of the whole sample genes was performed using the RunPCA function. In order to eliminate the batch effects, we integrated the samples through the “Harmony” R package. Finally, 17 PCA dimensions were selected and further clustered by FindNeighbors and FindClusters functions. According to the “singleR” R package and literature data, these groups were annotated. The mark gene expression was calculated in each cluster cell using the FindAllMarkers function. Meanwhile, we extracted the glandular epithelial cells and tumor cells from the cluster. Cell trajectory analysis was performed using the “monocle” R package. The change in gene expression of DR-DEGs in cell trajectories were also investigated. We further analyzed the expression of DR-DEGs in single-cell clustering and found that the GATA1 was mainly expressed in plasma B and mast cells.

### 2.8 Experimental Verification

#### 2.8.1 Experimental preparation

Cells (AN3-CA) used in the experiment were provided by Wuhan University Cell Bank; FBS and dmem were purchased from Gibco; 6-well plates were purchased from Corning; the Transwell Room was purchased from Corning; the CCK8 cytotoxicity kits were purchased from Toyobo Life Science, and capsaicin was purchased from Shanghai universal Biotech Co.,Ltd.

#### 2.8.2 Cell wound scratch assay

Cells were inoculated in a 6-well plate until fully covered. A wound was created with a 200ul gun head in the 6-well plate. The original medium was replaced with MEM serum-free medium containing 0 and 100 μM capsaicin, respectively. The wound area was photographed and measured at 0, 24 and 48 h.

#### 2.8.3 Transwell invasion assay

MEM complete medium 600 μL was added into the Transwell which was placed therein. AN3CA cells were diluted to 1 × 10^6^/ml with serum-free DMEM medium containing 0 and 100 μM of capsaicin, of which 100ul was respectively added into the different Transwell. After 24 h, the Transwell was fixed in pre-supercooled paraformaldehyde for 30 min. The Transwell was inverted on table. A drop of crystal violet solution was dropped in the Transwell and stained for 20 min. The crystal violet solution was washed off with PBS buffer solution after 20 min. The cells and medium inside the Transwell were completely gently wiped off with a cotton swab, then the Transwell was inverted on the table and dried. After that it was observed under an inverted microscope. The number of cells traversed in each group was observed under a 10× microscope for statistical analysis.

#### 2.8.4 CCK8 assay

AN3CA cells were inoculated in 96-well plates at a density of 5,000 cells per well and cultured in complete cell medium for 24 h for recovery. Next, the medium was changed to mem serum-free medium containing 0,100 um capsaicin and incubated for 24 h. Then, 10 μL of CCK8 solution was added to each well and incubated for a further 1 h at 37°C. The absorbance was measured at 450 nm using an enzyme marker.

#### 2.8.5 Quantitative-polymerase chain reaction tests

Cells were inoculated in a 6-well plate to 80% confluence. The original medium was replaced with MEM complete medium containing 0 and 100 μM capsaicin for intervention. RNA was extracted from blank group (MEM complete medium for 24 h) and drug group (DMEM complete medium containing 100 μM capsaicin) in six-well plates after 24 h according to the instructions of RNA Extraction Kit of Feijie Biological Company. CDNA was retrotranscribed using a High Capacity cDNA kit (Thermofisher). GAPDH was used as the endogenous control. The primers were purchased from Shanghai Sangon Biotech Co., Ltd. See Table 1 for the sequences. The PCR reaction system is as follows: 10 μL of Premix Ex Taq (loading dye Mix), each 0.5 μL of upstream and downstream primers, 1 μL of cDNA, and ddH_2_O supplemented to the total system 20 μL. Reaction conditions: 94°C initial denaturation for 5 min; 94°C denaturation for 30 s; 60°C annealing for 30 s; 72°C extension for 1 min, 72°C final extension for 5 min after 35 cycles to the end of the reaction, and observation.

**TABLE 1:** The primer sequences for real-time PCR.

Gene	Forward	Reverse
GATA1	TTG​TCA​GTA​AAC​GGG​CAG​GTA	CTT​GCG​GTT​TCG​AGT​CTG​AAT
GAPDH	GCA​CCG​TCA​AGG​CTG​AGA​AC	TGG​TGA​AGA​CGC​CAG​TGG​A

## 3 Results

### 3.1 Acquisition of uterine corpus endometrial carcinoma-related DR-differentially expressed genes from capsaicin

**FIGURE 1:**
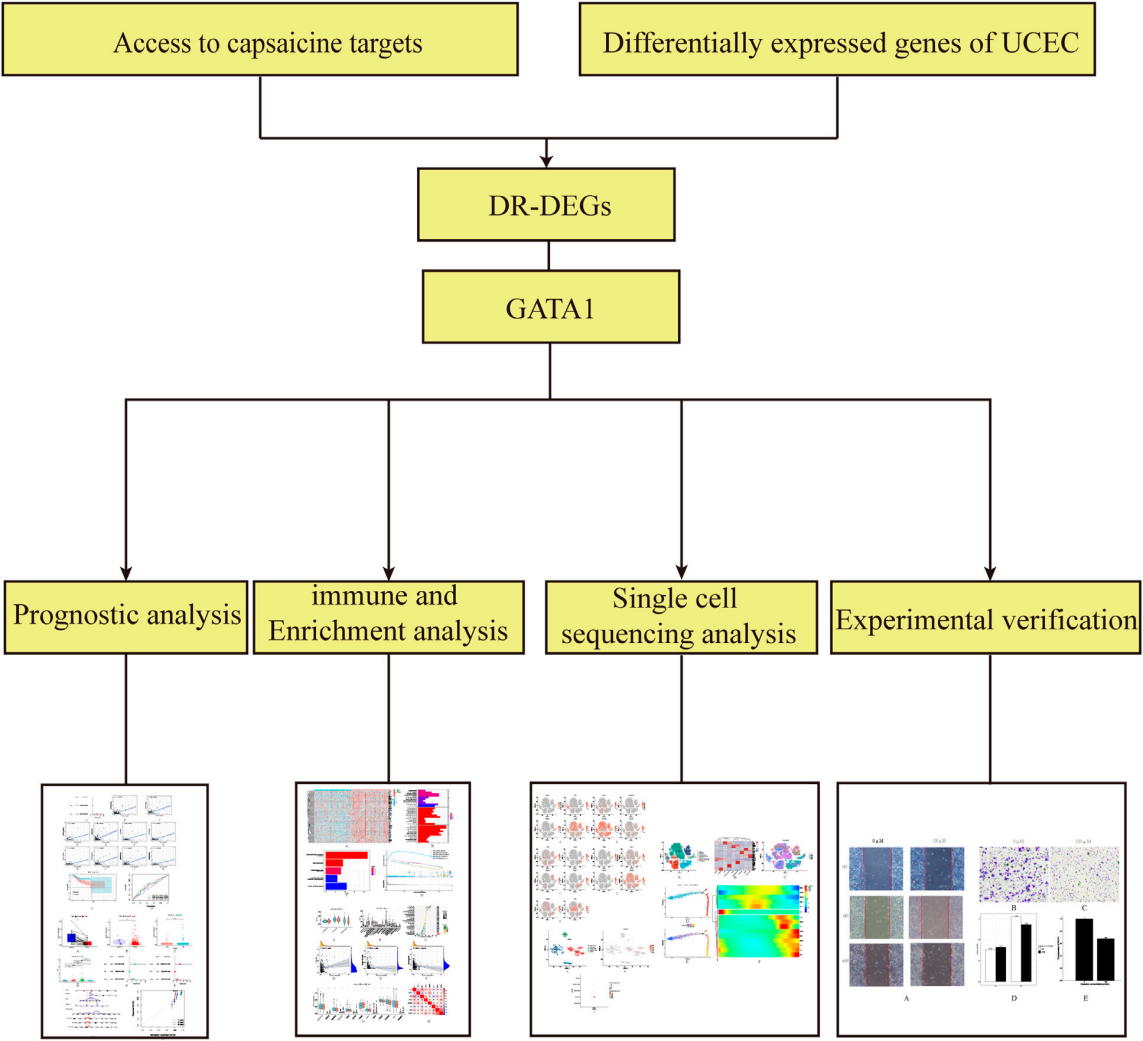
Workflow diagram for the design of this study.

**FIGURE 2:**
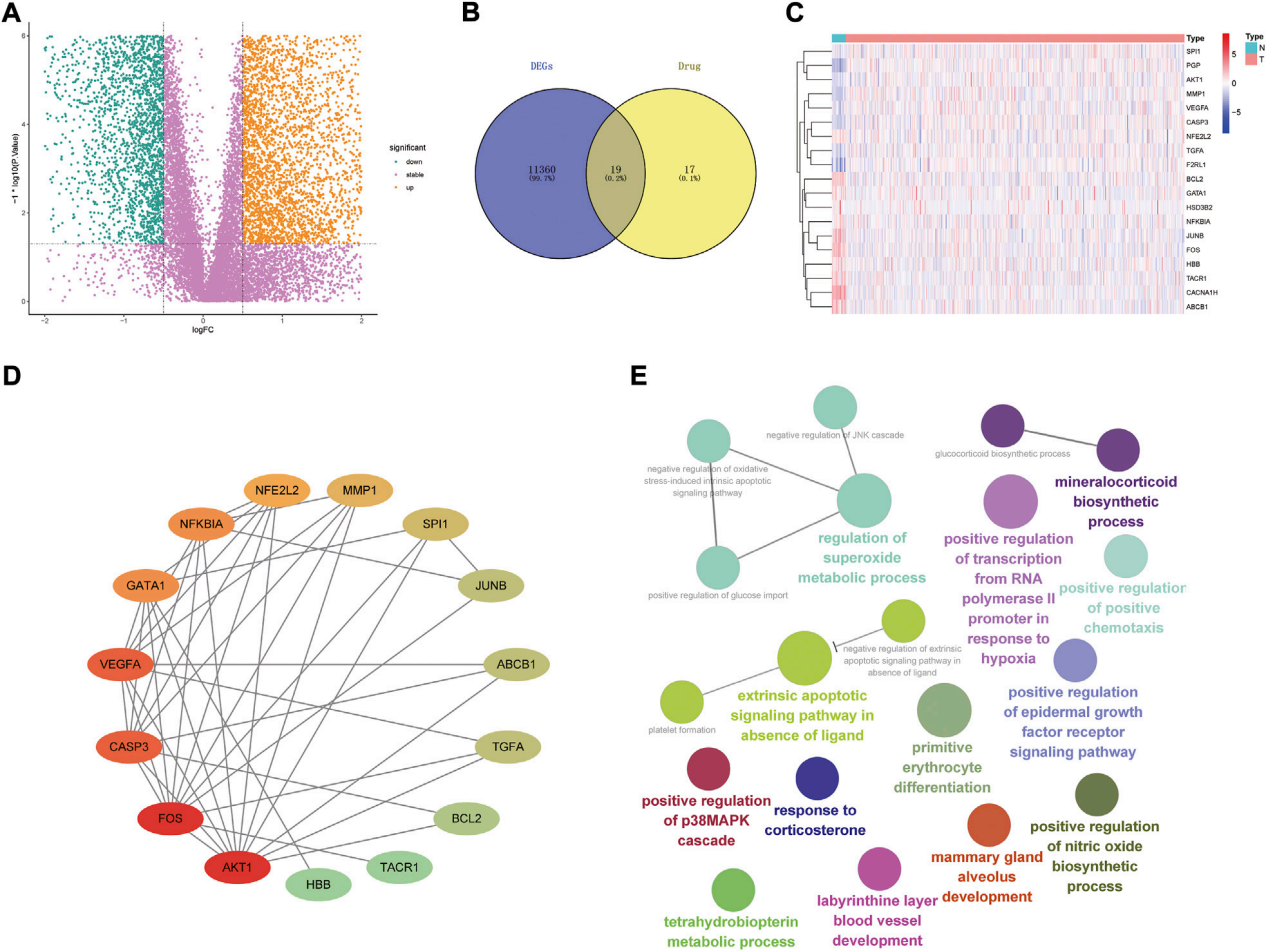
Interaction relationship and enrichment analysis of drug-associated differential genes; **(A)** Volcanoes of 6,989 endometrial cancer differential genes; **(B)** Venny diagram of 19 drug-associated differential genes; **(C)** Heat map of 19 drug-differential genes (N represents normal group, T represents tumor group; red represents high gene expression, blue represents low gene expression, the depth of colour represents high or low expression); **(D)** PPI protein interaction network relationship diagram of DR-DEGs (red represents high level of interaction, green represents low level of interaction; **(E)** Analysis of the enrichment effect of DR-DEGs.

The analysis flow chart is shown in Figure 1. By screening 522 differential genetic data related to UCEC from the tumor group versus 23 from the patients of normal group in the TCGA database, We obtained a total of 6,989 DEGs (Figure 2A). The intersection of 36 capsaicin target genes obtained from the TCMSP database and DEGs was taken place via the Venny 2.1.0 website. A total of 19 DR-DEGs (Figure 2B) were obtained. Its expression in the tumor and normal groups was also visualized by heatmap (Figure 2C). We found that eight genes were highly expressed in the tumor group and 11 genes were highly expressed in the normal group. Of GATA1 was highly expressed in the normal group. In order to explore the interaction of target proteins among DR-DEGs, we constructed a PPI network map of 19 DR-DEGs using the STRING (https://string-db.org/) database. The data were imported into Cytoscape (3.9.0) for further analysis and visualization (Figure 2D). The gene GATA1 was found to be highly correlated with target proteins, with seven genes related to the target proteins. Functional analysis of DR-DEGs was carried out using the Cytoscape (3.9.0) plug-in ClueGo (Figure 2E). It was found that the pathways of “regulation of superoxided metabolic process and extrinsic apoptotic signaling pathway in absence of ligand” Significant enrichment were significantly enriched.

### 3.2 Screening of prognosis-related genes

**FIGURE 3:**
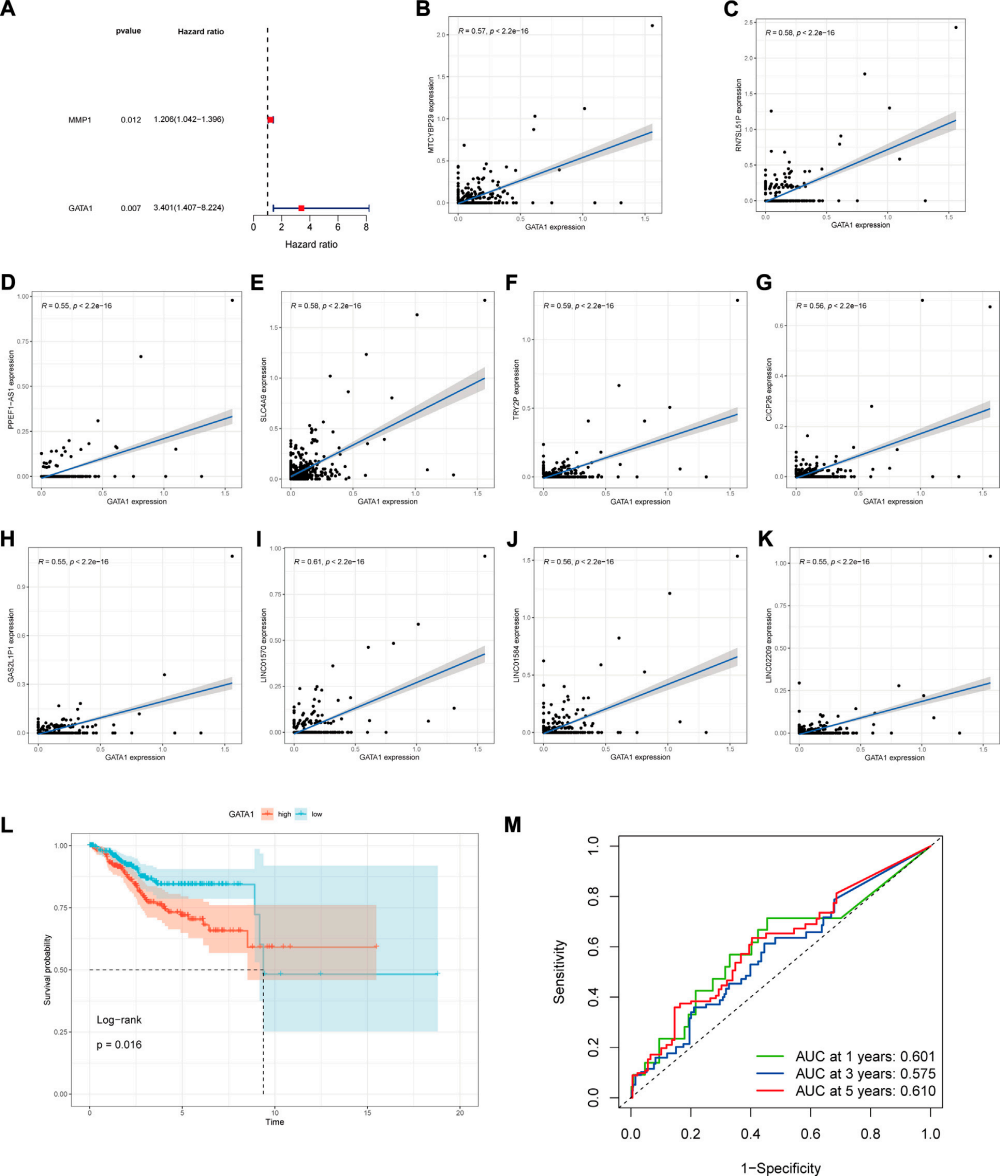
GATA1 prognosis and gene correlation analysis. **(A)** Forest plots of DR-DEGs associated with UCEC prognosis were obtained by univariate COX regression analysis; **(B–K)** GATA1 correlation with MTCYBP29, RN7SL51P, PPEF1-AS1, SLC4A9, TRY2P, CICP26, GAS2L1P1, LINC01570, LINC01584, LINC02209 correlation analysis; **(L)** Kaplan-Meier analysis plot of prognostic correlation between high and low GATA1 expression groups; **(M)** Time-dependent ROC plot.

Through the univariate Cox risk regression analysis of 19 DR-DEGs, we yielded two DR-DEGs related to prognosis (Figure 3A): MMP1 and GATA1, respectively. We detected that the GATA1 risk score was about 3 times that of MMP1 with a low *p*-value. Having combined with consulting relevant literature, we speculate that the GATA1 is more related to the prognosis of UCEC patients. Besides, it is a key target for the treatment of UCEC. Based on this, we used Pearsen to test the correlation of GATA1 and found that it was closely correlated with ten other genes (Figures 3B–K), including MTCYBP29, RN7SL51P, PPEF1−AS1, SLC4A9, TRY2P, CICP26, GAS2L1P1, LINC01570, LINC01584 and LINC02209, respectively. Moreover, they all have positive correlations. In order to explore the prognostic relationship between the GATA1 and UCEC patients, we counted the expression level of GATA1 in UCEC patients. UCEC patients were divided into high and low expression groups according to the median value, with 276 patients in the high expression group and 276 patients in the low expression group. The survival analysis was conducted between the high and low expression groups. The Kaplan-Meier curve showed that the 5-years OS was significantly higher in the low expression group than the high expression group (Figure 3L) (*p* < 0.05). The time-dependent ROC curve was used to evaluate the prediction effect (Figure 3M). The 1-year area under curve (AUC) was 0.601, 3-years AUC was 0.575, and 5-years AUC was 0.610. It indicated that the predictive effect was good and stable. A KM survival curve in the new supplementary validation group showed that the high expression of GALA1 was not conducive to a favorable prognosis for patients (*p* = 0.027) (Validation Supplementary Figure S1). According to the ROC curve, the 1-year, 3-years and 5-years ACU values were 0.586, 0.609 and 0.684, respectively (Validation Supplementary Figure S2). Our results are further supported by this finding.

### 3.3 Independent prognostic value of GATA and clinical correlation analysis

**FIGURE 4:**
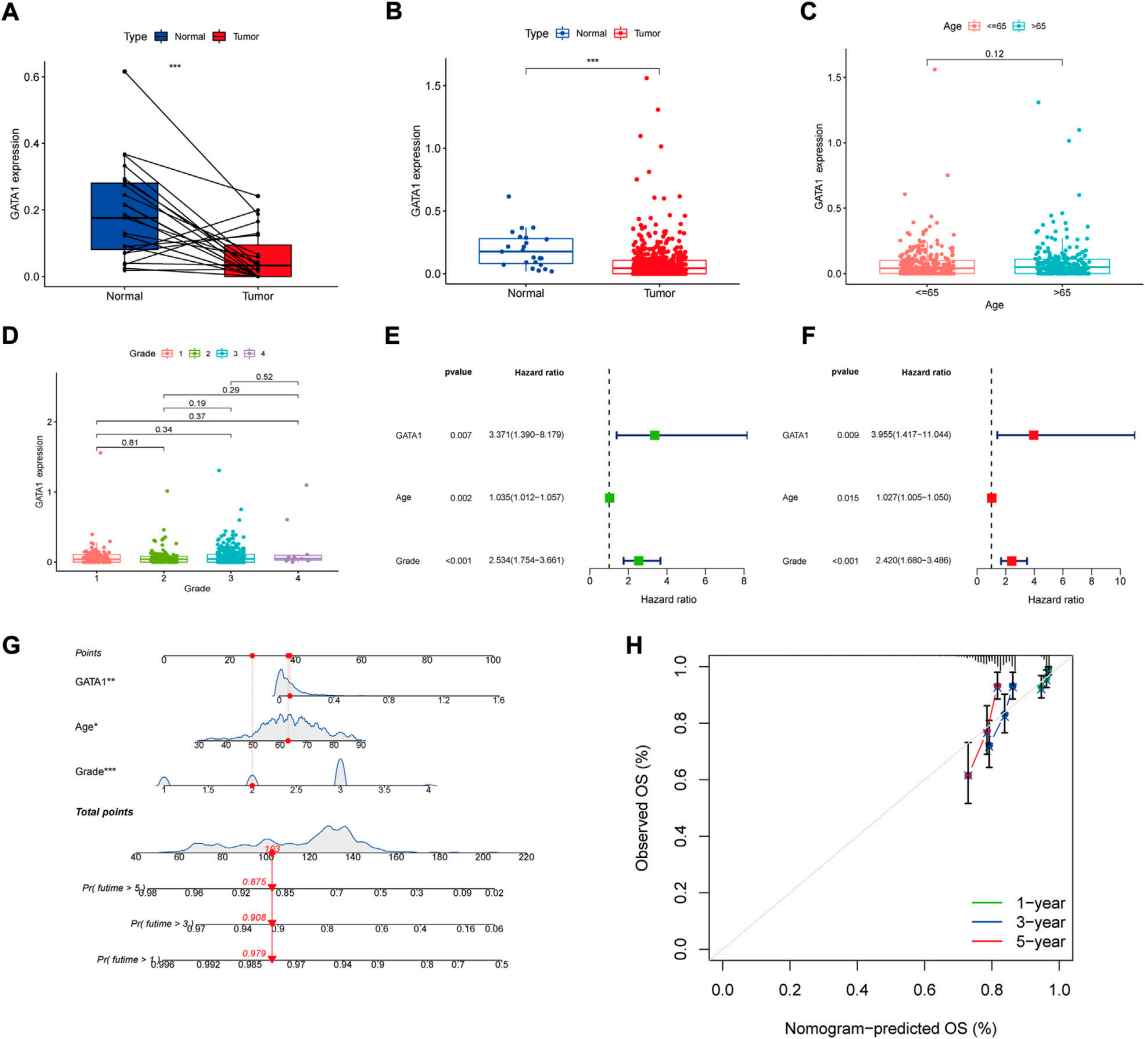
GATA1 clinical correlation and independent prognostic analysis. **(A)** GATA1 expression in patients in the tumor group and in tumor and normal tissues; **(B)** GATA1 expression in patients in the normal and tumor groups; **(C)** GATA1 expression in samples from patients in different age groups; **(D)** GATA1 expression in samples from patients at different stages; **(E,F)** Univariate and multivariate Cox regression independent prognostic analyses; **(G)** Nomogram predicting 1-, 3- and 5-years survival rates in UCEC patients; **(H)** Columnar plot calibration curves for testing deviations between predicted and actual 1-, 3- and 5-years survival probabilities in nomogram.

By comparing the GATA1 gene expression between the patients’ endometrial tumor tissue and normal tissues (Figure 4A), we found that the GATA1 expression was higher in the normal tissue than the tumor tissue. We further compared the GATA1 between the normal group and the tumor group, and found that the GATA1 expression was still high in the patients’ endometrial tissue of the normal group (Figure 4B). In order to investigate the correlation of GATA1 with clinical data, we separately analyzed the expression level of GATA1 in patients at the different ages and different tumor stages. According to the results (Figure 4C), GATA1 expression did not differ significantly between age groups. Furthermore, GATA1 expression was not significantly different between grades (Figure 4D). In order to evaluate whether the GATA1 could exist as an independent prognostic factor, we performed univariate and multivariate COX regression analyses for its relation with the age and tumor stage, respectively (Figures 4E,F). The results showed that the age, tumor stage and GATA1 could all exist as independent prognostic factors. Moreover, the GATA1 had the highest risk score. In the interest of better prediction of the survival rate of UCEC patients, we integrated the GATA1 and clinical factors to build a nomogram graph prediction model (Figure 4G). The total score was obtained by adding the score values of each variable together to predict the 1, 3 and 5-years survival of each patient. The calibration curve was drawn to test the accuracy of the model (Figure 4H). The results showed that the calibration curve fitted with the ideal curve. The predictive effect of the nomogram model was more accurate.

### 3.4 Functional enrichment analysis

**FIGURE 5:**
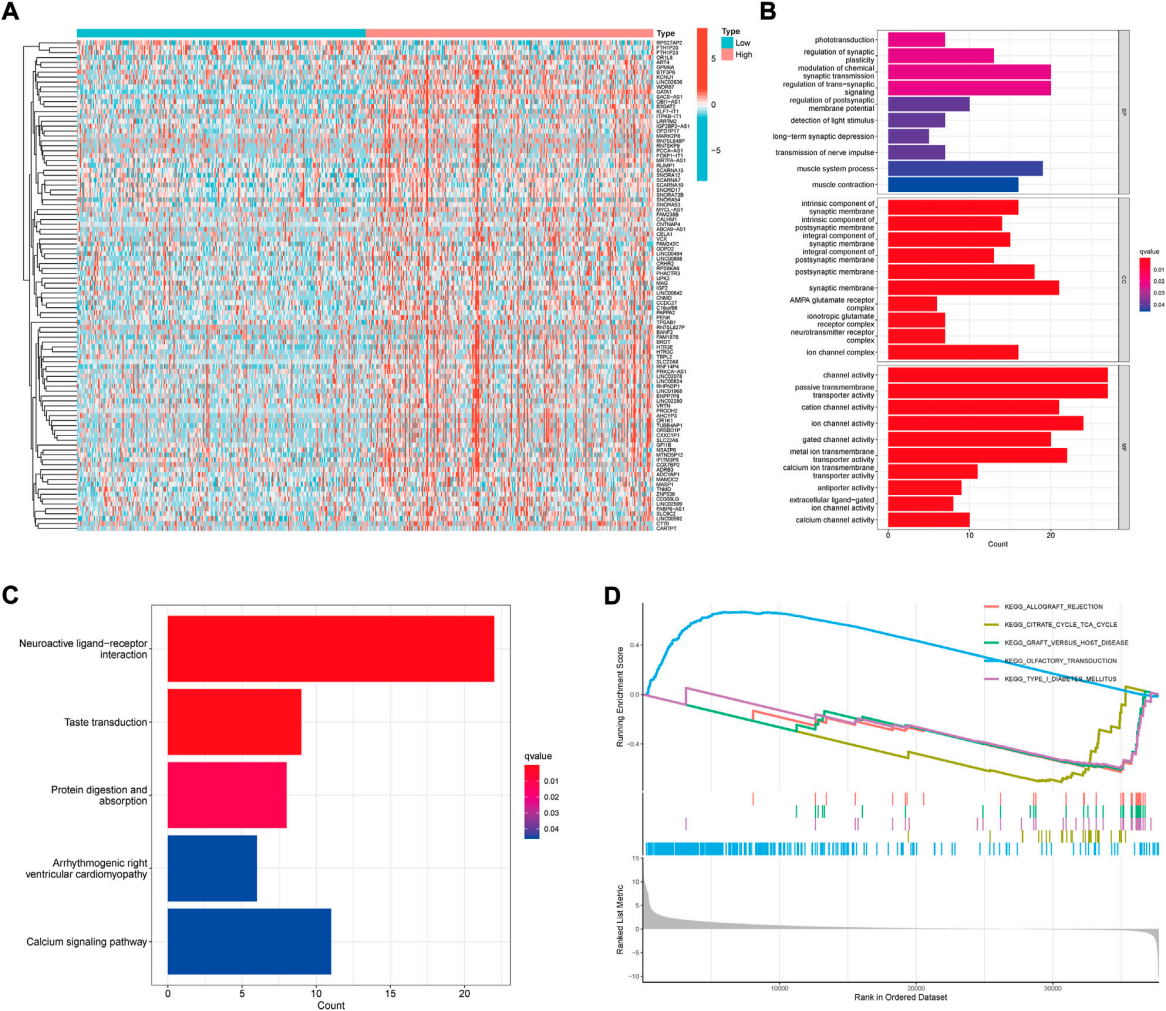
Functional analysis of differential gene enrichment in high and low expression groups. **(A)** Heat map of differential genes in high and low expression groups; **(B)** Functional analysis of GO enrichment; **(C)** Functional analysis of KGGG enrichment; **(D)** Functional analysis of GSEA enrichment.

In order to explore the potential pathways and mechanisms related to the GATA1 gene expression, we screened the significantly different genes in patients with high and low expression groups (Figure 5A). GO and KEGG enrichment analyses were performed for them. Of the GO analysis showed significant enrichment in the “Neuroactive ligand−receptor interaction” (Figure 5B) while the KEGG showed significant enrichment in the “integral component of synaptic membrane, regulation of postsynaptic membrane potential, synaptic membrane” (Figure 5C). GSEA gene enrichment analysis (Figure 5D) was further conducted on the gene sets of patients in the high and low risk groups. We found that the “OLFACTORY TRANSDUCTION ″ was significantly enriched in the high expression group while the “TYPE I DIABETES MELLITUS” was significantly enriched in the low expression group.

### 3.5 Tumor microenvironment and immunoassay

**FIGURE 6:**
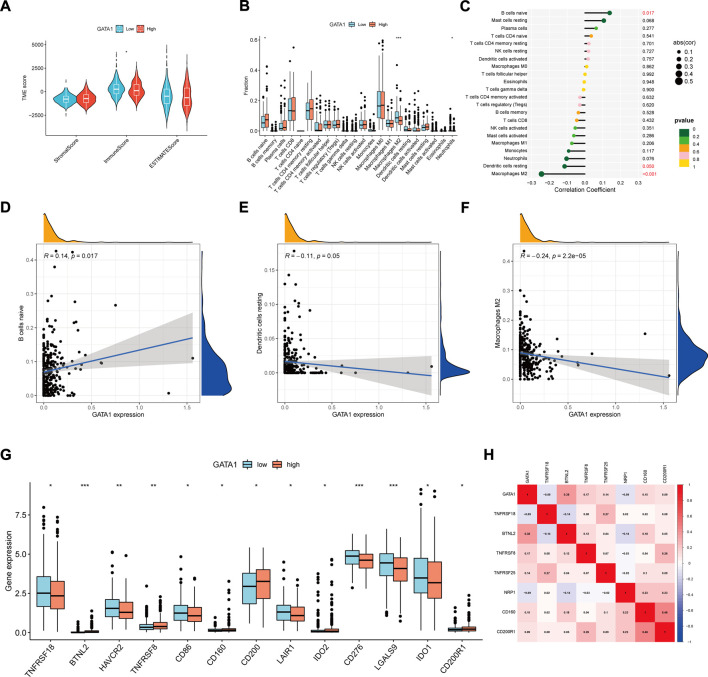
Tumor microenvironment and immune correlation analysis: **(A)** Distribution of stromal, immune, and total scores in tumor microenvironment between high and low expression groups; **(B)** Analysis of immune cell infiltration in high and low expression groups; **(C)** Correlation between GATA1 and immune cells for analysis; **(D–F)** Relationship between GATA1 and B cells naïve, Dendritic cells resting, and Macrophages M2 expression; **(G)** Box line plot of immune checkpoint gene scores in high and low expression groups; **(H)** Co-expression relationship between GATA1 and immune checkpoint genes.

Through the “estimate” R package, we calculated the stromal cells and immune cells (Figure 6A) of tumor microenvironment and their total scores in the high and low expression groups, and found that there was no significant difference between the total and matrix scores. However, the immune scores in the lower expression group were slightly higher than those in the higher expression group. In order to further investigate the potential relationship of GATA1 and immunity, we worked out the infiltration scores of highly and lowly expressed immune cells using the algorithm of CIBERSORT deconvolution (Figure 6B). It was found that macrophages M2 scored higher in the low expression group while B cells naive scored higher in the high expression group. On this basis, we analyzed the correlation between the GATA1 and immune cells (Figure 6C). It was found that the B cells naive, dendritic cells resting and macrophages M2 were significantly correlated with the GATA1 expression. Of B cells naive showed a positive correlation (Figure 6D) while dendritic cells resting and macrophages M2 showed a positive correlation (Figures 6E,F). By analyzing the expression of immune checkpoint genes in the high and low expression groups (Figure 6G), we found that most genes were highly expressed in the low expression group while CD200 was highly expressed in the high expression group. The co-expression relationship of GATA1 and immune checkpoint genes (Figure 6H) was further analyzed, we found that it was negatively correlated with TNFRSF18, NRP1 and positively correlated with other genes.

### 3.6 Single-cell sequencing and correlation analysis of capsaicin target genes

**FIGURE 7:**
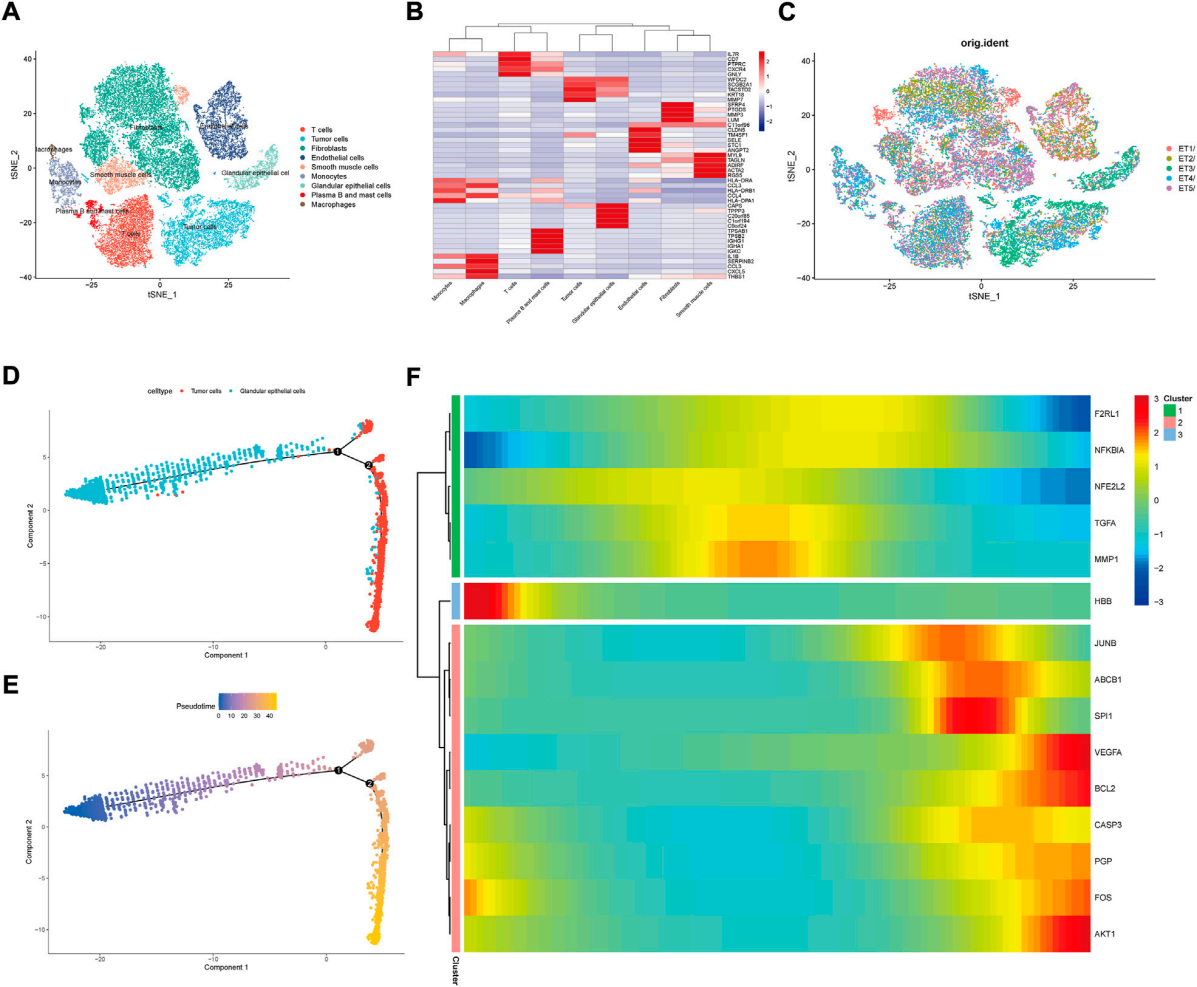
Single-cell sequencing subgroups and cell trajectory analysis. **(A)** Nine major cell subgroups of UCEC tumor tissues were constructed by t-SNE analysis; **(B)** Visualization of Marker gene expression in the 9 cell subgroups; **(C)** Distribution of single-cell sequencing data from five UCEC patient samples in t-SNE analysis; **(D,E)** Glandular epithelial cells versus Tumor cells in cell trajectory analysis graphs; **(F)** Expression changes of DR-DEGs with cell trajectory.

**FIGURE 8:**
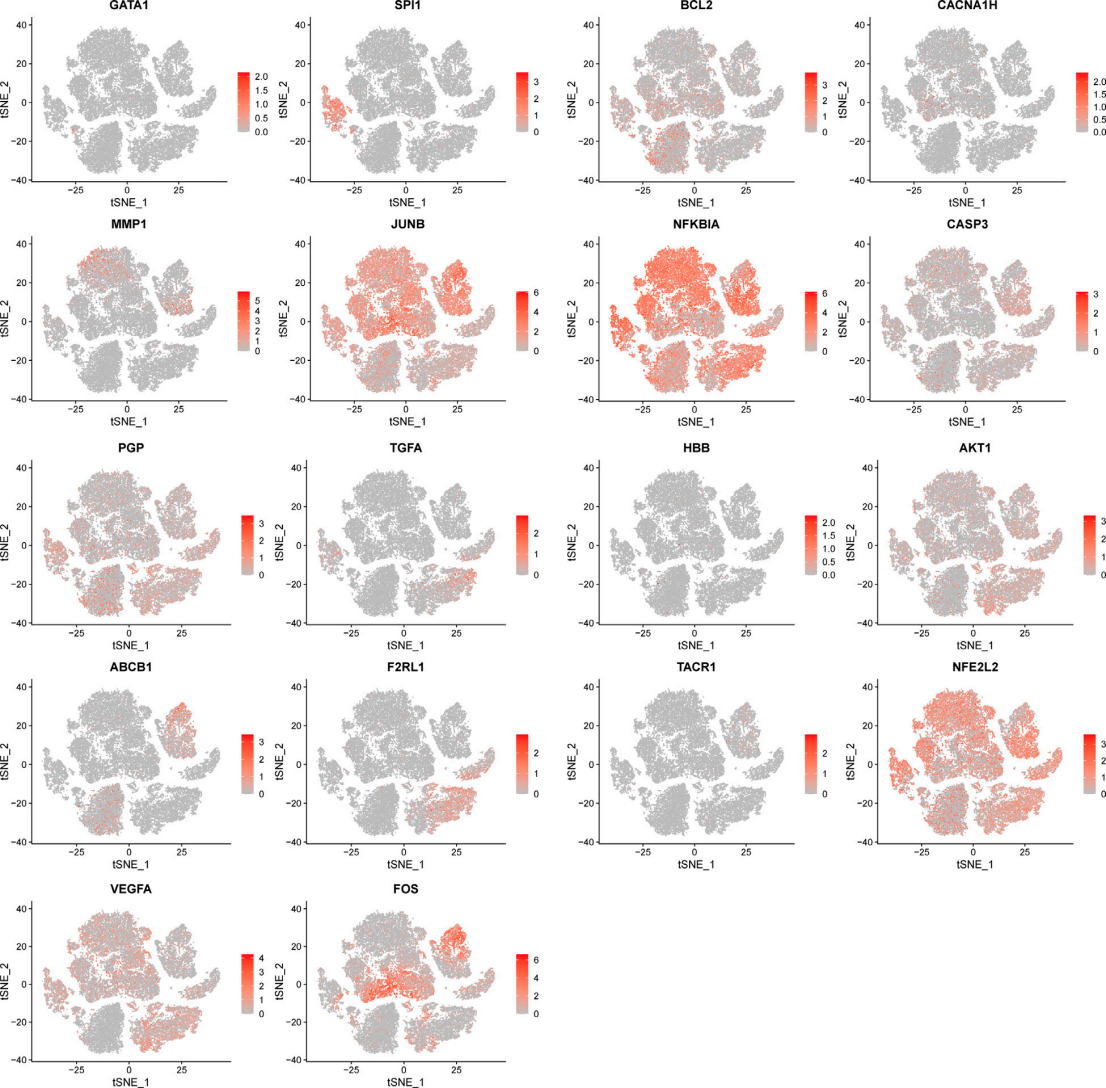
Distribution of t-SNE expression of DR-DEGs in single cell sequencing data.

**FIGURE 9:**
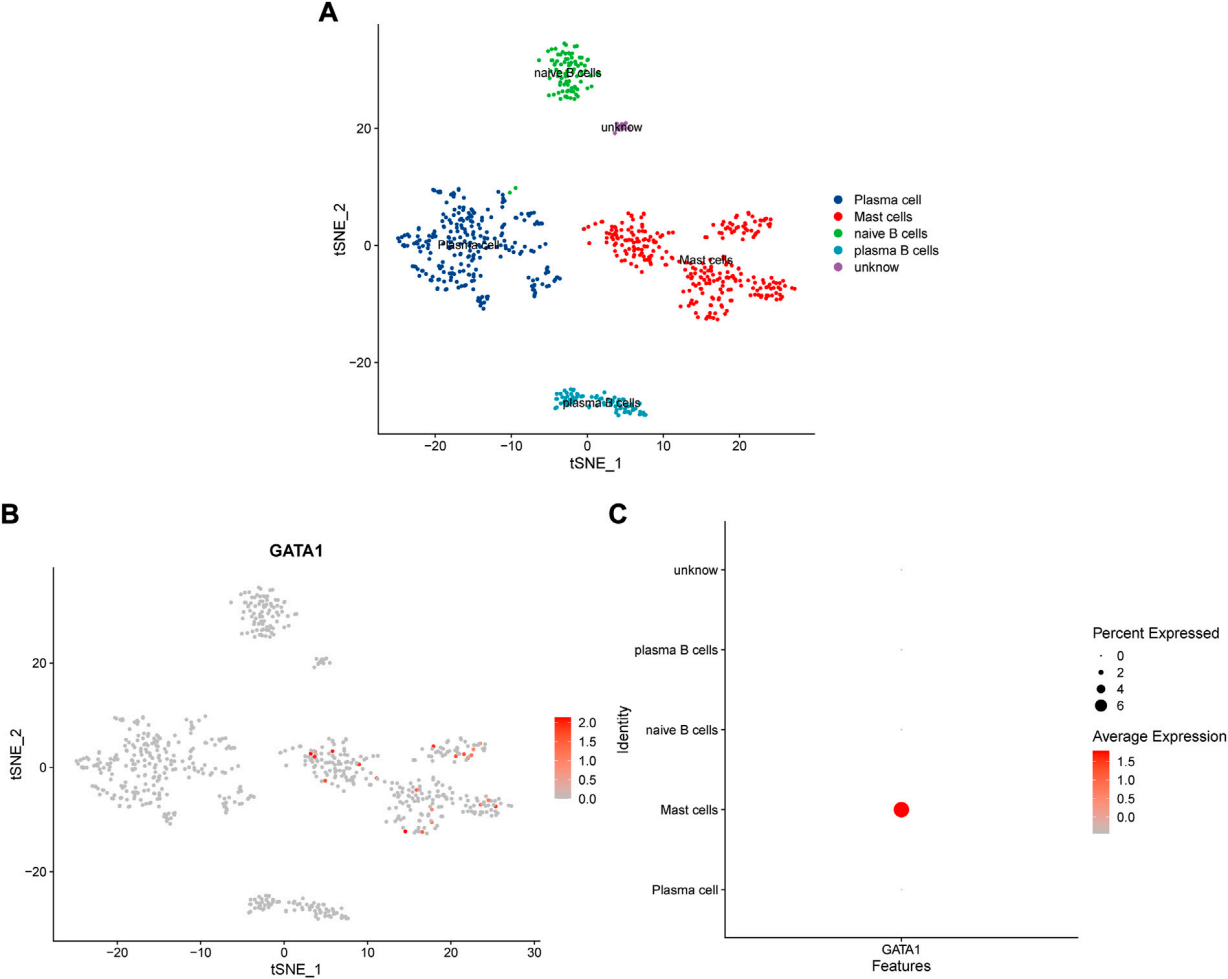
Expression relationship of GATA1 in cell grouping of Plasma B and mast cells; **(A)** Further clustering of Plasma B and mast cells into different subgroups; **(B,C)** Expression of GATA1 in different subgroups of Plasma B and mast cells.

We downloaded a total of five single-cell sequencing samples of UCEC patients from the GEO database (Supplementary Figure S1), screened and filtered them using R software (Supplementary Figure S2), and finally obtained and analyzed 34,210 cell sequencing data. In order to validate whether the gene expression within the cell stages has affected the analysis, we performed testing by the CellCycleScorin function (Supplementary Figure S3), and found that the number of different stage points in the PCA chart was relatively concentrated, indicating that the cell stages had few impacts on the analysis results. The dimensionality reduction of all sample genes was carried out by RunPCA function (Supplementary Figure S4). The FindNeighbors and FindClusters functions were further used to cluster them into 24 groups (Supplementary Figure S5). These 24 groups were annotated through the “singleR” R package and reviewing literature. They were eventually clustered into 9 classes of cells, including T cells, tumor cells, fibroblasts, endothelial cells, smooth muscle cells, monocytes, glandular epithelial cells, plasma B and mast cells and macrophages (Figure 7A). The expression of MARK genes in different cell groups was calculated by FindAllMarkers function (Figure 7B). The distribution of five samples of UCEC patients was investigated in the cluster (Figure 7C). We found that all five samples were distributed in the different groups. Whereas the ET3 patients were more significantly distributed in the glandular epithelial cells and tumor cells groups. For purpose of exploration of the relationship between the glandular epithelial cells and tumor cells, we conducted a cell trajectory analysis (Figures 7D,E) and found that tumor cells could be developed from glandular epithelial cells. The change in DR-DEGs expression was further explored in the cell trajectory (Figure 7F). We found that the HBB expression might gradually decrease with cell trajectory, while VEGFA, BCL2, CASP3, PGP, FOS and AKT1 gene expression could gradually increase with cell trajectory. We further explored the expression of DR-DEGs in the UCEC single-cell sequencing (Figure 8), and found that GATA1 was significantly expressed in plasma B and mast cells. In order to investigate the interrelationship between the plasma B, mast cells and the GATA1, we further clustered and annotated the plasma B and mast cells, and found that it could be divided into five categories (Figure 9A), namely plasma cells, mast cells, naive B cells and plasma B cells. For the remaining category, we cannot determine what kind of cells they belong to. We further investigated the expression relationship between the GATA1 and the new cell groups, and found that the GATA1 was mainly expressed in mast cells (Figures 9B,C).

**FIGURE 10:**
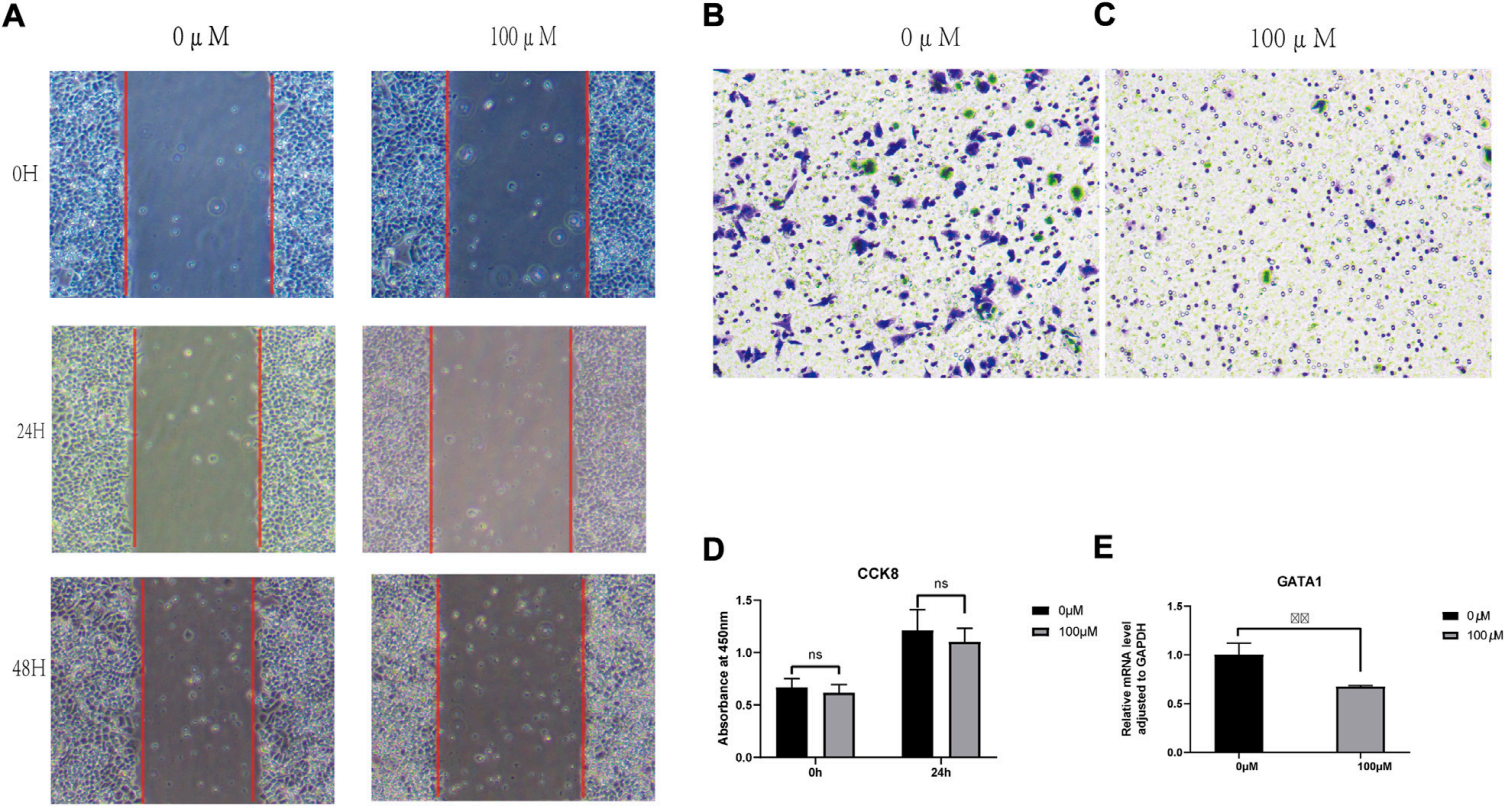
Experimental verification. **(A)** Wound Healing Assay; **(B,C)** Transwell assay; **(D)** CCk8 cytotoxicity test; **(E)** q-PCR.

### 3.7 Experimental Results

#### 3.7.1 Scratch assay

Wound-healing assays were performed to assess the effect of capsaicin on an3ca cell migration. The results showed that the difference in migration distance was significant (*p* > 0.05) between the 0 and 100 μmol/L capsaicin treatment groups. Moreover, the cell migration ability was poor under 100um of capsaicin treatment (Figure 10A).

#### 3.7.2 Transwell assay

Twenty-four hours after different concentrations of capsaicin acting on AN3CA cells, the results showed a significant difference in the number of transmembrane cells in AN3CA cells when the concentration of capsaicin was at 0 and 100 μM, respectively. Compared to the blank mem group, the number of transmembrane an3ca cells, t was significantly decreased under 100um capsaicin treatment (Figures 10B,C).

#### 3.7.3 CCK8 results

The wells with or without capsaicin were tested for cck8 at 0 and 24 h of stimulation. It could be seen that there was no statistical difference between the two groups at 0 h, proving that the cell status of the two groups was basically the same, and the test was repeated after 24 h. It was found that the cck8 absorbance of the 100 um capsaicin group was significantly lower than that of the 0 um capsaicin group, but it had not yet reached the half lethal concentration (IC50) (Figure 10D).

#### 3.7.4 Quantitative-polymerase chain reaction results

Compared with the blank MEM group, the expression level of gata1 RNA decreased significantly (*p* < 0.05) in the capsaicin-treated experimental group (Figure 10E). The difference was significant.

## 4 Discussion

As a common tumor in the female reproductive system with increasing morbidity and mortality in recent years, UCEC has become an important factor affecting women’s life and health (Urick and Bell, 2019). At present, the UCEC is mainly treated by surgery or in combination with adjuvant methods such as radiotherapy and chemotherapy at the advanced stage (Braun et al., 2016; van den Heerik et al., 2021). Although the early UCEC patients have good prognosis currently, those advanced and metastatic UCEC patients usually have poor prognosis (Weiderpass et al., 2014). Moreover, retaining their fertility through non-surgical therapy is particularly important for premenopausal UCEC patients (Westin et al., 2021). Therefore, it is particularly important to find new drugs and potential targets for treating UCEC, as well as to construct a new prognostic model. As a characteristic spicy compound in red pepper, capsaicin’s beneficial effects in the human body have been extensively studied. It plays an important role in easing pain, anti-inflammation, cardiovascular protection, anticancer and anti-obesity, etc. (Brederson et al., 2013) Nevertheless, the relevant role of capsaicin in UCEC has not been deeply explored. In this study, we have used transcriptome sequencing and single-cell sequencing data analysis to study the relevant role and potential mechanism of capsaicin in UCEC, discover biomarkers and build a prognostic model. Finally, it is validated by cell experiments.

Through univariate and multivariate Cox regression analyses of DR-DEGs, we have finally confirmed that MMP1 and GATA1 are correlated with prognosis, of which the GATA1 risk score is about three-fold that of MMP1. In combination with the literature review, we believe that the GATA1 is closely related to the prognosis and treatment of UCEC. It is possible to become a new therapeutic target. GATA1 is a key transcription factor in erythropoiesis, and controls multiple cell cycles such as cell survival, differentiation and apoptosis. Its mutation or deletion can lead to a variety of hematological diseases (Kerenyi and Orkin, 2010; Crispino and Horwitz, 2017). Research findings reveal that the GATA1 is also associated with poor progression and prognosis of cancer. For example, Yu et al. have found that the GATA1 may also promote the proliferation and metastatic invasion of colorectal cancer cells by activating the P13K/AKT pathway (Yu et al., 2019). GATA1 also plays a poor prognostic role in female tumors. It has been found that the GATA1 may enhance the invasive effect of tumors by enhancing the expression of survivin in the anti-apoptosis protein family. It may also induce proliferation and growth of tumor cells by activating HNF1A-AS1 transcription. Both are related to the progression and poor prognosis of breast cancer (Boidot et al., 2010; Yang et al., 2021). Liu et al. have also discovered that the GATA1 may induce the proliferation, migration and invasion of ovarian cancer cells by controlling and regulating JAG1-Nocth (Liu et al., 2020). By calculating the survival rates and survival curves of the high and low GATA1 expression groups, we have found that the survival rate of the high GATA1 expression group is low, indicating that the GATA1 may also be an unfavorable factor for the survival of UCEC patients. This is consistent with the results reported in the literature above for GATA1. Interestingly, after determining GATA1 as a high-risk gene, we have also found that the GATA1 expression is lower in the tumor tissue than the normal tissue. By reviewing literature, we have discovered that the low GATA1 expression is associated with poor prognosis of clear renal cell carcinoma and recurrence of lymph node metastasis (Peters et al., 2015). Some studies reveal that the GATA1 transcription is associated with the generation of immune-related cells, such as eosinophils, mast cells and dendritic cells (Yu et al., 2002; Kitamura et al., 2006; Scheenstra et al., 2016). We hypothesize that the low level of GATA1 expression in tumor tissues may be related to the UCEC and some immune functions. Based on this, we have performed a further analysis and validation on the immunization and GATA1.

Under immune cell infiltration analysis, we have found that macrophages M2 has a higher score in the low expression group while B cells naive has a higher score in the high expression group. However, by reviewing literature, we have found that the GATA1 does not express in B cells and T cells (Ling et al., 2018). Moreover, because the differential expression in most immune cells are not significantly in the high and low expression groups, we have therefore further performed the tumor microenvironment and immune checkpoint analysis. We have discovered that the immune scores of lower expression group in the tumor microenvironment are slightly higher than those of higher expression group. The difference in other scores is not significant. Based on this, we have also performed an immune checkpoint analysis. The immune checkpoints are receptor-ligand pairs related to immune function, including stimulating immune checkpoint molecules and inhibitory immune checkpoint molecules (Zhang and Zheng, 2020). Both enhancing stimulating immune checkpoints and blocking inhibitory immune checkpoints can be used in the treatment of tumors (Zhang and Vignali, 2016; He and Xu, 2020). Among them, we have found that the immune checkpoint scores are generally lower in the high expression group while CD200 and CD200R are higher in the high expression group. The CD200 is a membrane protein and widely expressed in a variety of cells such as lymphocytes, nerve cells and endothelial cells. It may act as a bone marrow cell receptor ligand and play a role of inhibiting signaling (Wright et al., 2000; Barclay et al., 2002). It may also regulate the immune signals through CD200R and suppress the lymphocyte-mediated immune response (Gorczynski et al., 1999; Moreaux et al., 2008). Existing studies have found that CD200 is highly expressed in a variety of tumors, such as melanoma, myeloma and gastric cancer (Moreaux et al., 2006; Petermann et al., 2007; Zgodziński et al., 2018). It can also cooperate with CD200R to exert its inhibitory anti-tumor immunomodulatory function and promote the tumor growth and development. Based on this, we speculate that GATA1 expression is high in UCEC tumor tissue while the survival decreases. In addition to enhancing cancer cell invasion, it may be associated with immune function suppression. However, except for the inhibitory immune checkpoint genes CD200 and CD200R have higher scores in the high expression group, the other checkpoint genes have lower scores in the high expression group. This is the same as our point of view. However, its further potential functions and mechanisms still need to be further validated by experiments and clinical practice.

In order to further explore the underlying mechanism of GATA1 in the treatment of UCEC, we have performed functional analysis of DEGs in the high and low expression groups. In the KEGG enrichment analysis, we have found that the DEGs are significantly enriched on neural function-related neuroactive ligand-receptor interaction pathways. Studies have demonstrated that neuroactive ligand-receptor interaction is correlated with cancer progression and metastasis, such as gastric cancer, osteosarcoma and colorectal cancer (Muff et al., 2015; Yao et al., 2021; Yu et al., 2021). Chronic stress is a high risk factor for cancer occurrence and development. However, the chronic stress is found to have an effect on the neuroactive ligand receptor interaction enrichment pathways and drive the progression of cancer (Le et al., 2016; Lei et al., 2021). Pressure and stress may also activate the sympathetic nervous system and lead to disorders of the neuroendocrine system, promote inflammation and tumor vascularization, leading to the growth of tumor cells and spreading to nearby tissues (Sloan et al., 2010; Kim-Fuchs et al., 2014). Meanwhile, we have also found significant enrichment on its neurosynaptic function-related pathways in GO, such as“integral component of synaptic membrane, regulation of postsynaptic membrane potential and synaptic membrane”. Thus, capsaicin may act on UCEC through the modulation of neural function. By using the GSEA enrichment analysis, we have found that the pathway “TYPE I DIABETES MELLITUS” is significantly enriched therein. It has long been found that diabetes is one of the risk factors for UCEC (Brinton et al., 1992). A study on 9,000 cancer patients in type 1 diabetes has found that the incidence of UCEC is significantly higher in the general population (Carstensen et al., 2016). Studies have found that the probability of UCEC in women with type 1 diabetes is about 42% higher than that of normal people (Wise, 2016). In addition, a significant increase is also observed in mortality in UCEC patients with type 1 diabetes (Harding et al., 2015). Therefore, this mechanism may be related to the treatment and prognosis of UCEC. We believe that controlling blood sugar may reduce the probability of the occurrence and death of UCEC. However, the specific mechanism still needs to be further investigated.

Through single-cell sequencing correlation analysis, we have found that the distribution of the patient sample ET3 is more significant in the glandular epithelial cell group and tumor cell group. The intercellular time trajectory relationship is further investigated in the two groups. Moreover, the expression of gene HBB gradually decreases with the progression of glandular epithelial cells to tumor cells. HBB is one of the members of the globin family, a component of globin in the hemoglobin chain. The heme group therein gives red cells a sufficient force to transport oxygen (Giardina et al., 1995; Bonaventura et al., 2013). In addition to being expressed in erythroid cells, it also expresses in pulmonary epithelial cells, macrophages and mesangial cells (Liu et al., 1999; Bhaskaran et al., 2005; Nishi et al., 2008). Onda et al. have found that the HBB expression generally decreases in thyroid cancer line cells, and the growth of thyroid cancer line cells could be significantly inhibited by forced HBB expression (Onda et al., 2005). Maman et al. have found that the HBB and its derivative Metox can effectively inhibit the metastasis of tumor cells. Furthermore, it down-regulates the ERK phosphorylation and induces tumor cell apoptosis and cell cycle arrest through TAK1 and P38 (Maman et al., 2017). It plays an important role in inhibiting the transformation of normal cells into tumor cells, thereby suppressing the tumor progression. Interestingly, we have found that the capsaicin could specifically stimulate the expression of the HBB genes (Lee et al., 2007). Thus, combined with the results of the sequencing analysis, we speculate that the capsaicin could exert an effect of HBB on cell cycle arrest in tumors by acting on HBB target genes, so as to inhibit the progression of glandular epithelial cells to tumor cells and achieve the purpose of treating UCEC. By further clustering, we have found that the GATA1 is mainly expressed in mast cells. Mast cells are derived from bone marrow hematopoietic stem cells and are granular congenital immune cells, which may cause an inflammatory response through the release of various inflammatory factors (Ammendola et al., 2013; Ammendola et al., 2014; Ammendola et al., 2016; Andersen et al., 2016). The long-term low-level inflammatory environment is considered one of the favorable conditions for tumor growth (Crusz and Balkwill, 2015). Whereas the leukotriene, prostaglandin and histamine secreted by mast cells may enhance and promote the persistence of the inflammatory response (Oskeritzian, 2015), thus having a promoting effect on tumor growth. Mast cells may also participate in the hydrolysis of the extracellular matrix and endothelial cell basement membrane by secreting matrix metalloproteinase (MMP), thus contributing to the remodeling of the tumor microenvironment and enhancing the invasion ability of tumor cells (Komi and Redegeld, 2020), as well as promoting the tumor neovascularization in combination with these produced trypsin, growth factors and other angiogenic factors (Blair et al., 1997; Huang et al., 2008). Meanwhile, the trypsinase secreted by mast cells is found to promote the growth of blood vessels in UCEC tumor tissues to promote the tumor progression (Ribatti et al., 2005). Moreover, the GATA1 plays an important role in the growth and development of mast cells. Several studies have found that the progenitors responsible for differentiating mast cells are more susceptible to apoptotic by knocking down GATA1 in mice (Kitamura et al., 2006). Moreover, mature mast cells not only decrease in number, but also exhibit various abnormal forms (Migliaccio et al., 2003). Therefore, we speculate that mast cells play a promoting role in the occurrence and development of UCEC, while the GATA1 is responsible for the differentiation and maturation of mast cells. By inhibiting GATA1 expression, mast cell maturation could be suppressed in the tumor tissue, thus treating cancer. Interestingly, we have learned from cellular experiments that the GATA1 expression is significantly decreased in a group of capsaicin-treated cells. Therefore, we propose that the capsaicin may play a therapeutic role in the treatment of UCEC by targeting GATA1 expression and inhibiting the differentiation and maturation of mast cells.

Moreover, advances have been made in the study of prognostic markers including Long non-coding RNAs (LncRNAs), which have been associated with the immune and tumor microenvironments, as potential prognostic markers and therapeutic targets (Yuan et al., 2021). LncRNAs also have considerable prospects in UCEC. According to Liu et al. (Liu et al., 2021), genomic unstable long noncoding RNAs and immune-associated long noncoding RNAs may have prognostic value for UCEC. Whereas we chose GATA1, the therapeutic target of natural medicine small molecules, as the study object, and further explored its mechanism through single-cell analysis and experiments. As a result, this study provides a possible prognostic model for UCEC, as well as a possible and feasible direction for the treatment of UCEC using natural small molecules.

## Challenges and limitations

According to the literature, further *in vitro* and *in vivo* experiments are required to validate the hypothesis. Since there are no relevant clinical trial results, it has not been possible to determine whether there is a definite therapeutic effect on UCEC. The aim of more studies will be to investigate and verify the specific therapeutic effects of capsaicin on UCEC *in vivo* and in clinical randomized controlled trials.

## Conclusion

As a small molecule of TCM with anti-inflammation, analgesia, weight control and anti-tumor effects, capsaicin’s therapeutic effect in UCEC has not been deeply studied. In this study, we have constructed a new prognostic model by acquiring the prognostic gene GATA1 of capsaicin associated with UCEC. On this basis, the treatment mechanism of capsaicin-related genes in UCEC is further explored by using the enrichment analysis and immune methods as well as in combined with the single-cell sequencing data. Finally, it is validated by cell experiments. Overall, our study however provides a new direction for the treatment of UCEC, also supplies new genetic markers for the prognosis of UCEC, as well as offers the basis for the development of capsaicin as a drug treatment for the UCEC.

## Data Availability

The datasets presented in this study can be found in online repositories. The names of the repository/repositories and accession number(s) can be found in the article/Supplementary Material.
