# Supplementary figures and images for: Mechanism investigation and experiment validation of capsaicin on uterine corpus endometrial carcinoma
Source: Front Pharmacol. 2022 Sep 21;13:953874. doi: 10.3389/fphar.2022.953874 (PMC9532580; doi:10.3389/fphar.2022.953874)

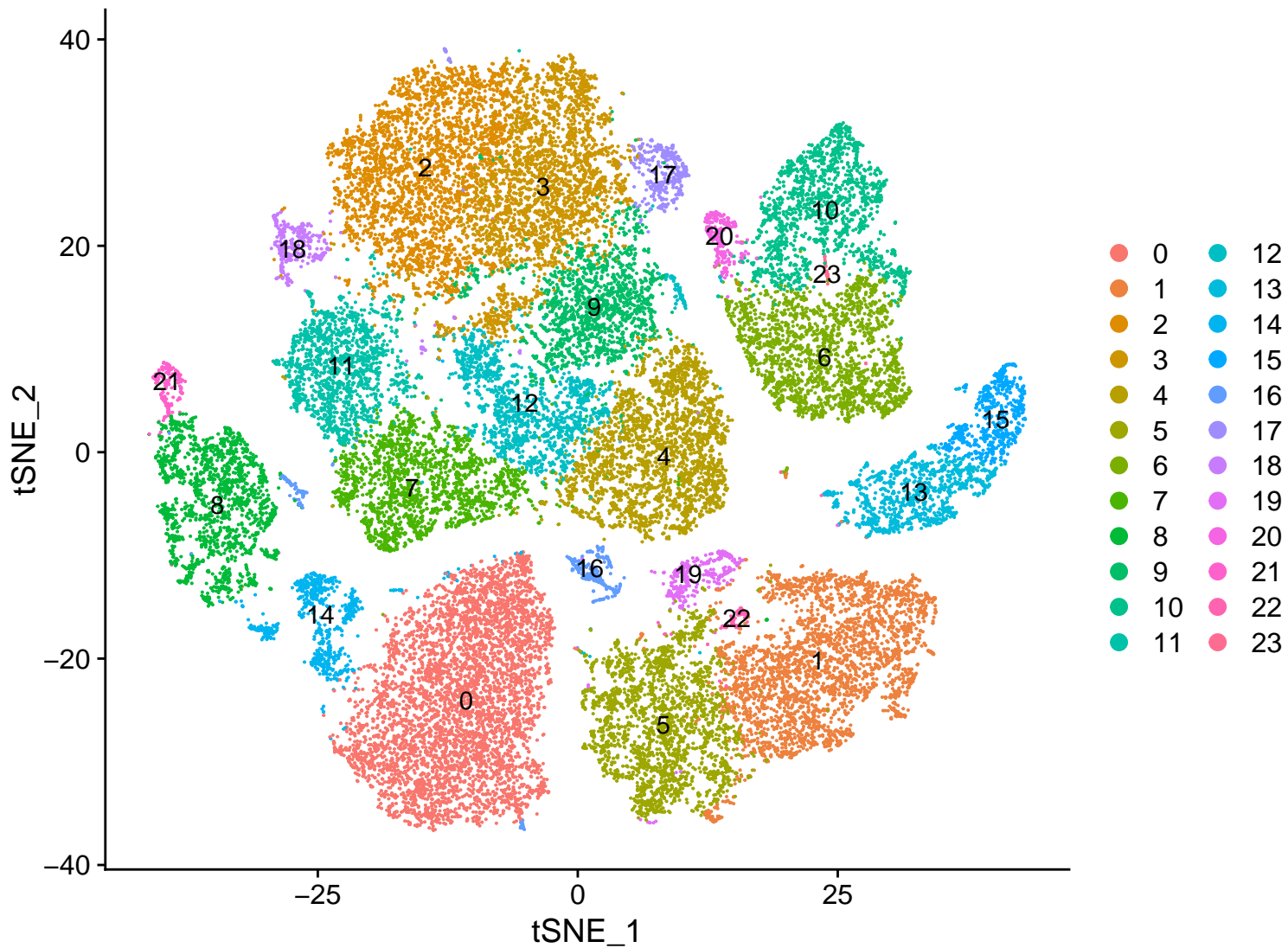

Supplement: Supplementary file 2 [file Image5.PDF]

GATA1 high low

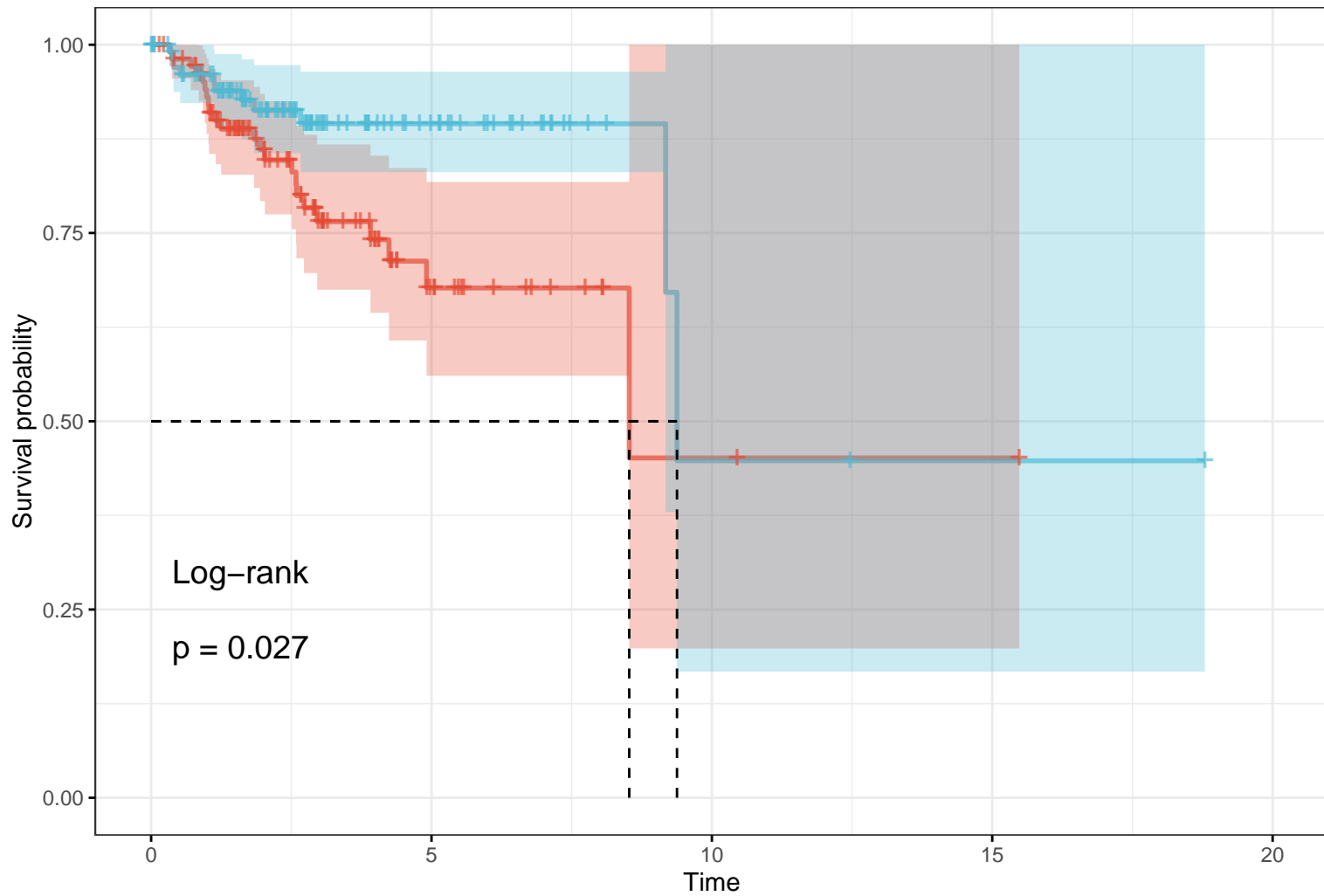

Supplement: Supplementary file 3 [file Image6.PDF]

orig.ident

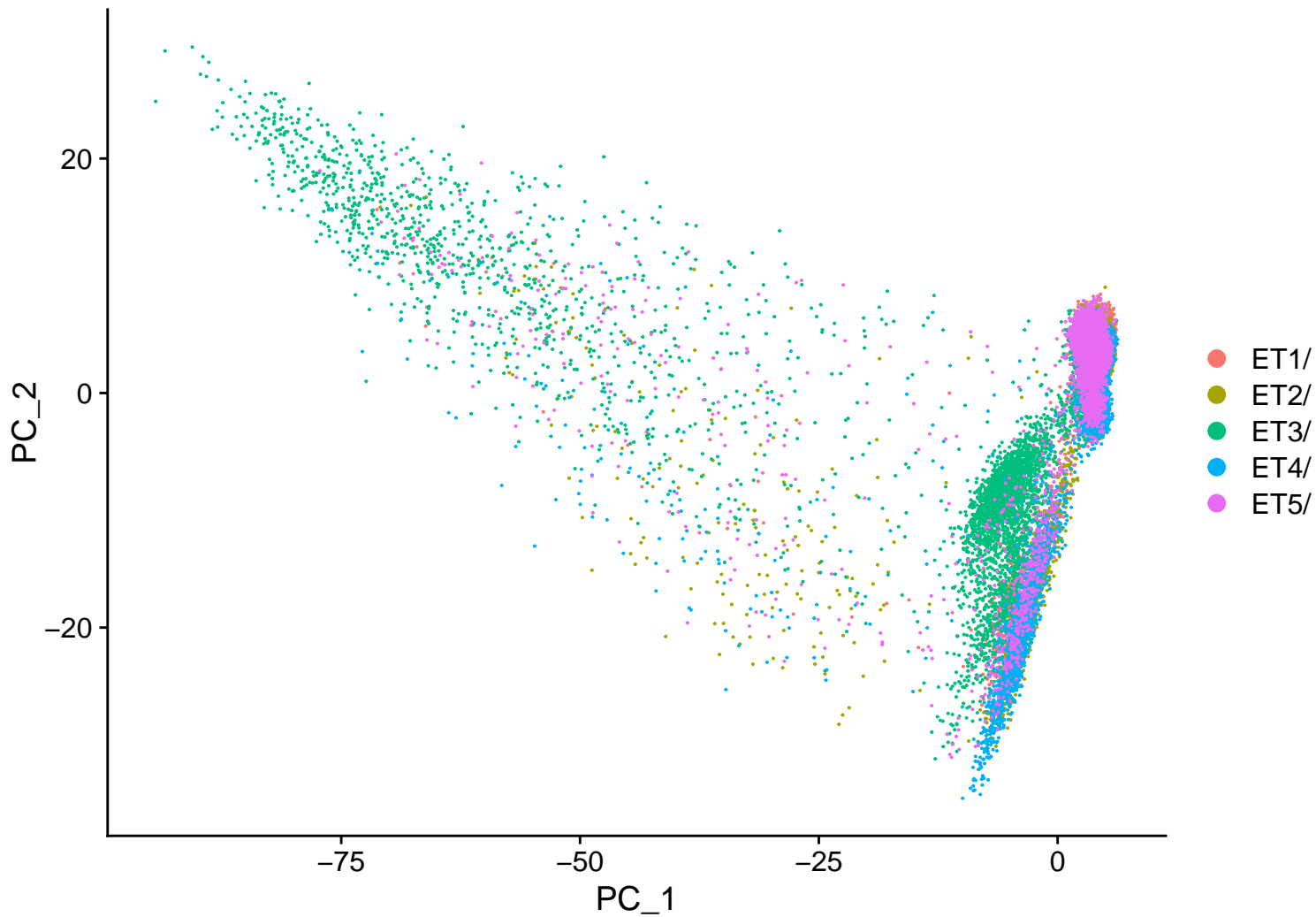

Supplement: Supplementary file 5 [file Image4.PDF]

**nFeature\_RNA**

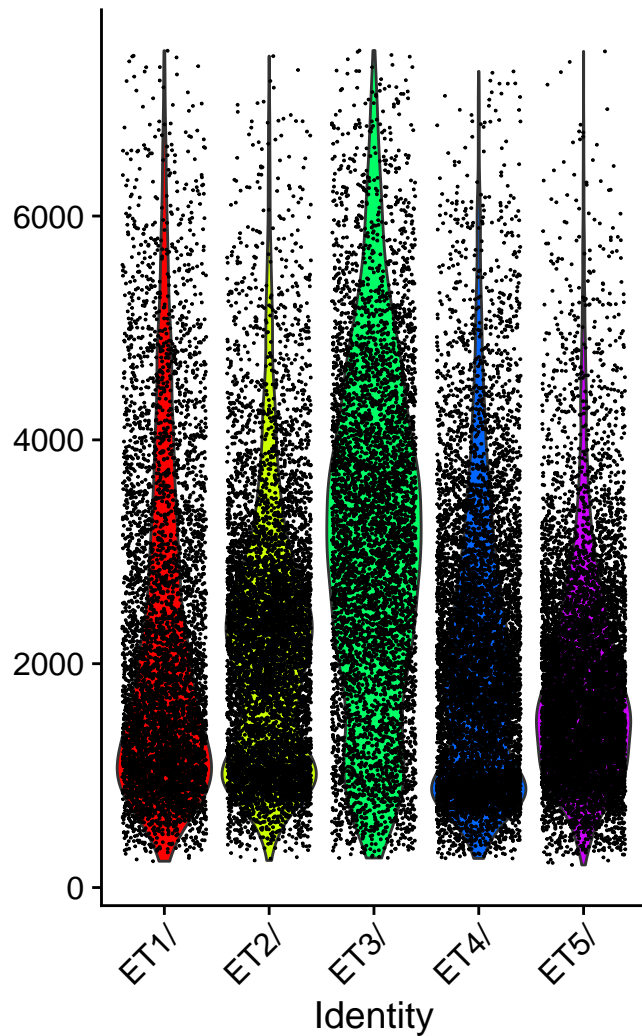

**nCount\_RNA**

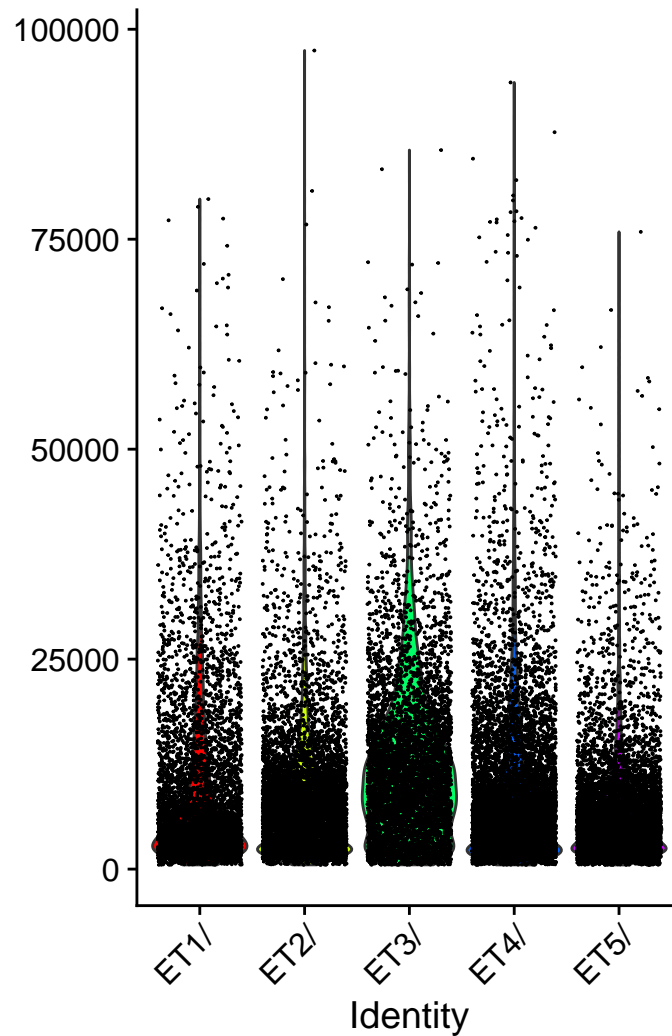

**percent.mt**

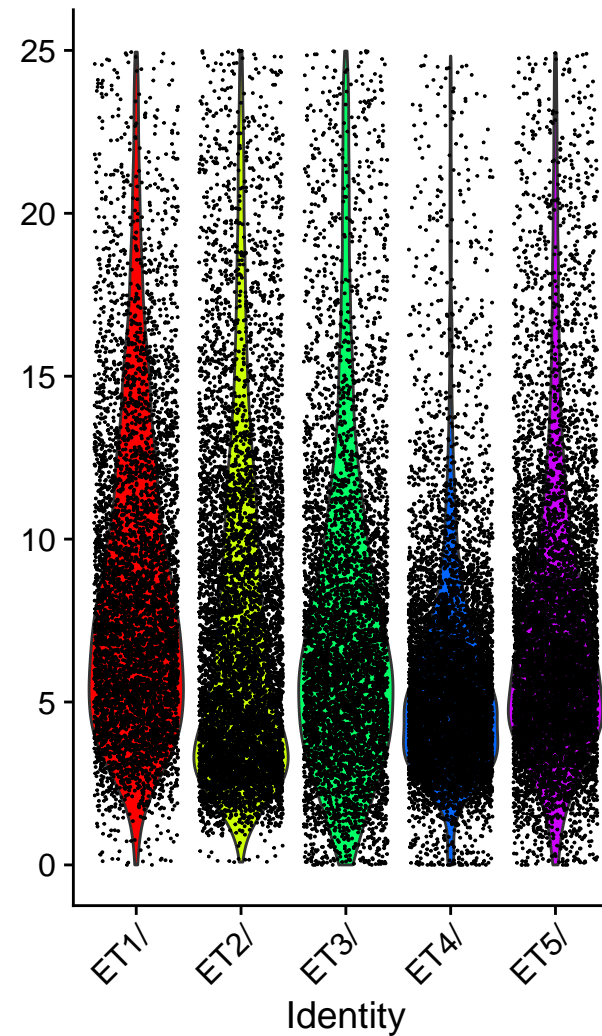

**percent.HB**

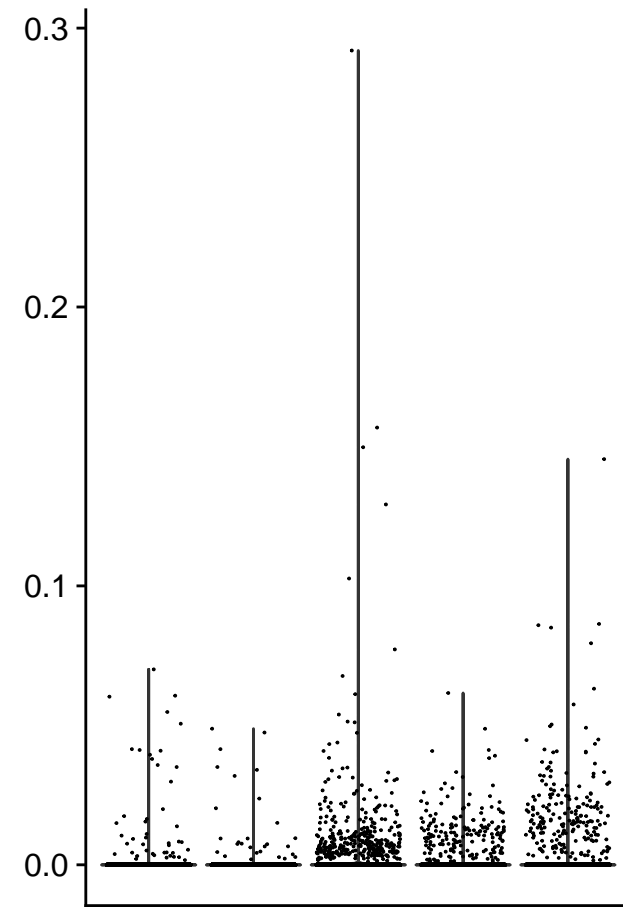

Supplement: Supplementary file 6 [file Image2.PDF]

# Phase

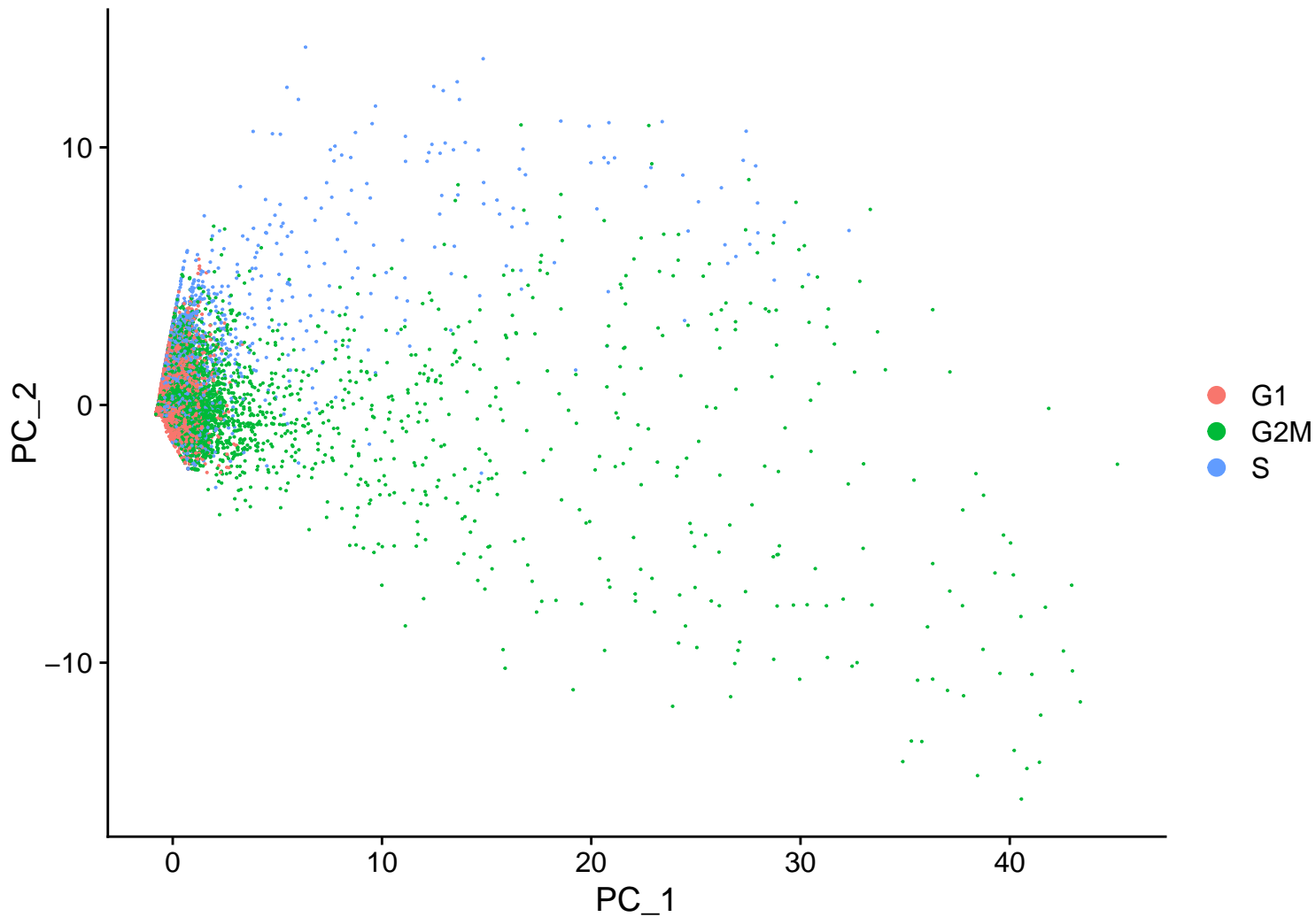

Supplement: Supplementary file 7 [file Image3.PDF]

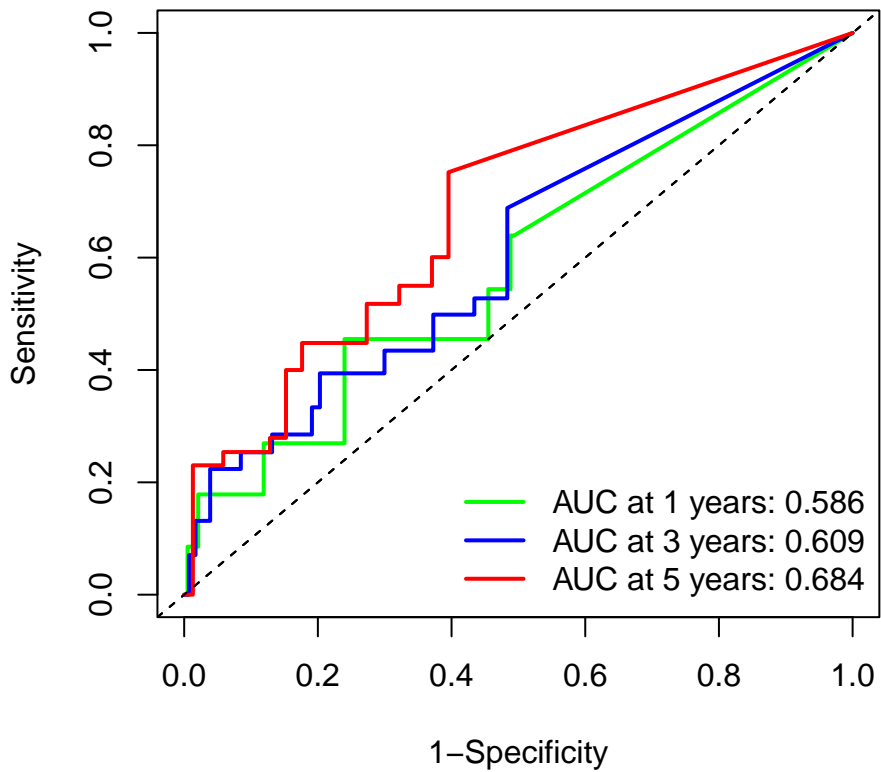

Supplement: Supplementary file 9 [file Image7.PDF]

**nFeature\_RNA**

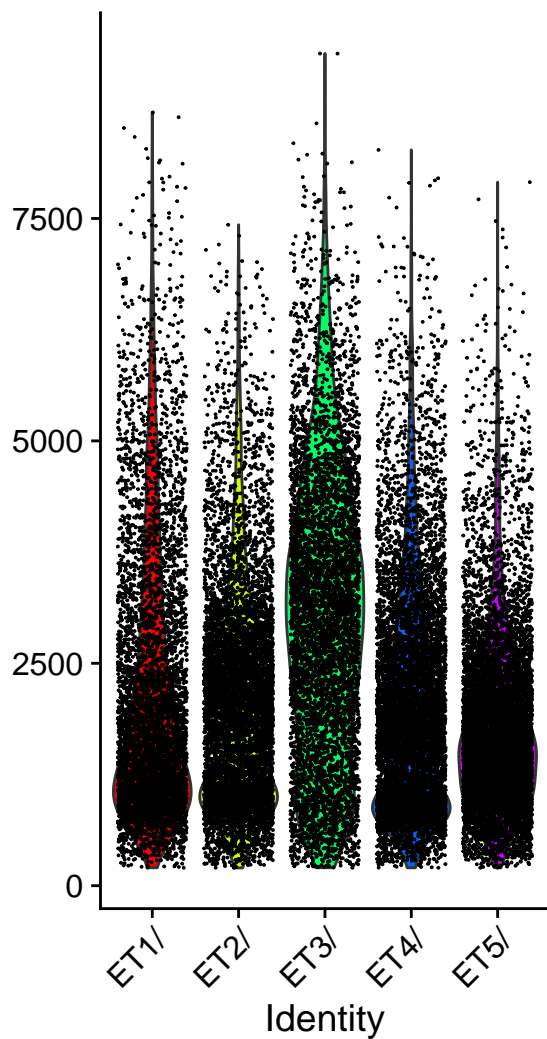

**nCount\_RNA**

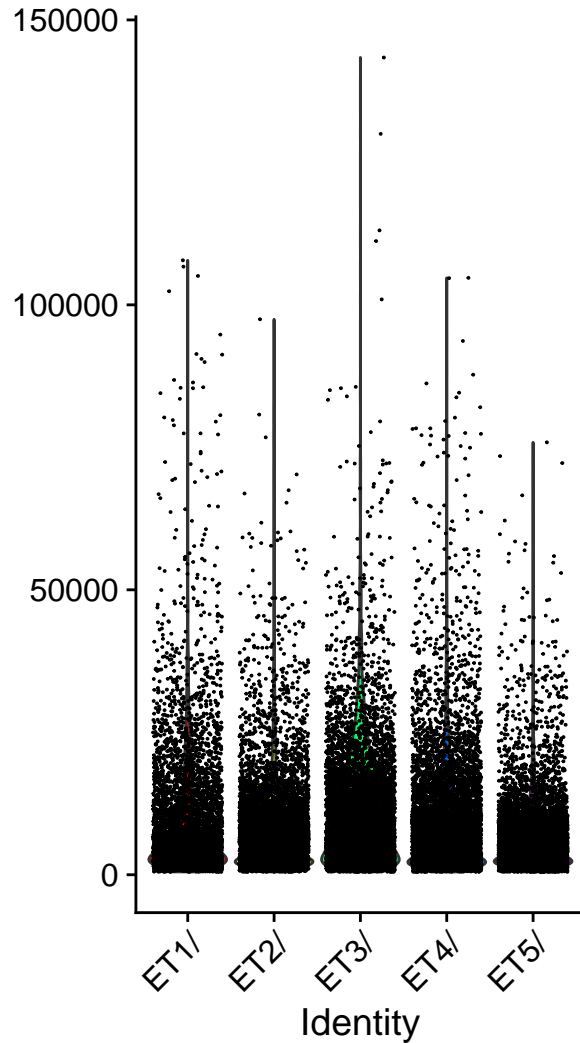

**percent.mt**

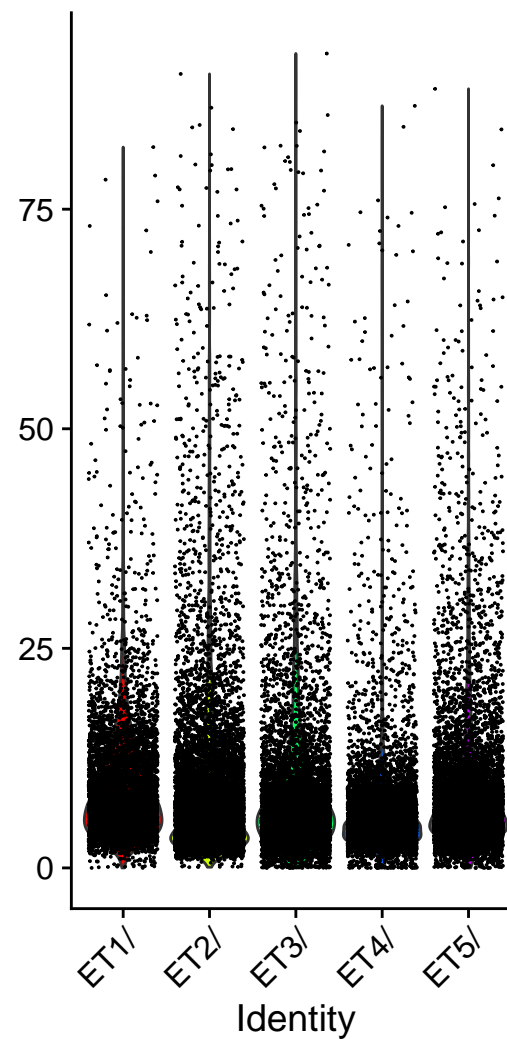

**percent.HB**

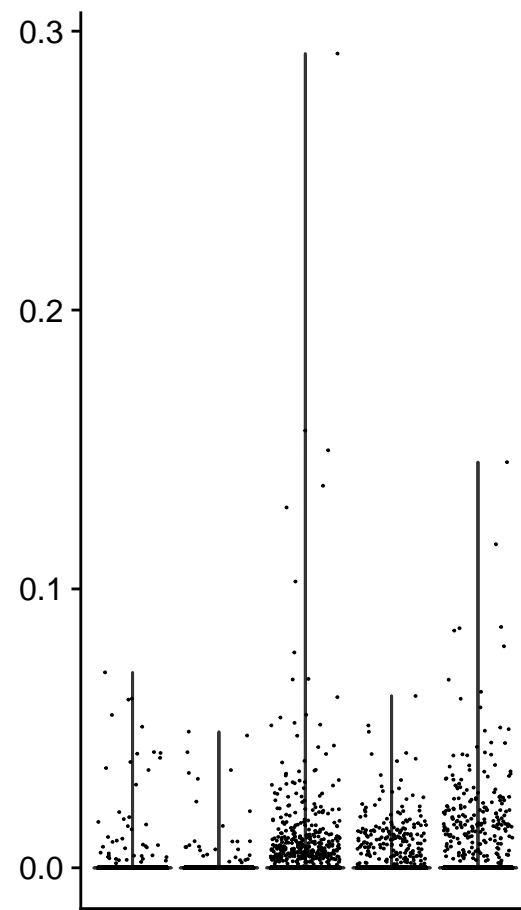

Supplement: Supplementary file 12 [file Image1.PDF]
